# A Survey of Advanced Border Gateway Protocol Attack Detection Techniques

**DOI:** 10.3390/s24196414

**Published:** 2024-10-03

**Authors:** Ben A. Scott, Michael N. Johnstone, Patryk Szewczyk

**Affiliations:** 1 School of Science, Edith Cowan University, Perth, WA 6027, Australiap.szewczyk@ecu.edu.au (P.S.); 2 School of Science, Engineering & Technology, RMIT University, Ho Chi Minh City 700000, Vietnam

**Keywords:** anomaly detection, BGP, cyber security, Internet security, routing security

## Abstract

The Internet’s default inter-domain routing system, the Border Gateway Protocol (BGP), remains insecure. Detection techniques are dominated by approaches that involve large numbers of features, parameters, domain-specific tuning, and training, often contributing to an unacceptable computational cost. Efforts to detect anomalous activity in the BGP have been almost exclusively focused on single observable monitoring points and Autonomous Systems (ASs). BGP attacks can exploit and evade these limitations. In this paper, we review and evaluate categories of BGP attacks based on their complexity. Previously identified next-generation BGP detection techniques remain incapable of detecting advanced attacks that exploit single observable detection approaches and those designed to evade public routing monitor infrastructures. Advanced BGP attack detection requires lightweight, rapid capabilities with the capacity to quantify group-level multi-viewpoint interactions, dynamics, and information. We term this approach advanced BGP anomaly detection. This survey evaluates 178 anomaly detection techniques and identifies which are candidates for advanced attack anomaly detection. Preliminary findings from an exploratory investigation of advanced BGP attack candidates are also reported.

## 1. Introduction

The Internet has been described as a complex, dynamic, massively distributed, scaled, and internetworked system whose designers could not have foreseen its evolution into one of humankind’s most profound creations [1,2,3]. Internet traffic is exchanged via connections using an inter-domain protocol [4]. The critical, and insecure, inter-domain protocol that binds the Internet is known as the Border Gateway Protocol (BGP); it connects more than 80,000 Autonomous System (AS) networks (henceforth referred to as ASs) by routing traffic between them. The number of ASs used may be larger when taking into account private and reserved ASs. Internetwork protocol security was not the primary focus for the designers of the Internet, although network protocol innovation is inextricably linked to the history of the Internet. For example, the Transmission Control Protocol and Internet Protocol (TCP/IP), which remains central to networking today and was developed from the work at the Defense Advanced Research Projects Agency (DARPA) [5].

Both malicious and non-malicious BGP incidents have affected major Internet entities including Akamai, Amazon, Apple, Facebook, Google, Mastercard, and Microsoft [6,7,8]. BGP anomalies can range in impact from the comparatively harmless example of route flapping through to destructive route leaks and hijacks [6,9,10]. BGP incidents are categorized as being either direct (intentional or unintentional), indirect, or outages [11]. BGP hijacking and route leaking incidents are well-established examples of direct incidents (e.g., the Telekom Malaysia incident). Whereas indirect events include cyber incidents that disrupt critical Internet operations and indirectly impact the BGP (e.g., Internet worms such as Wannacrypt). Outages are those stemming from natural disasters and/or energy system failures (e.g., the Moscow Blackout incident).

Despite the correlation of multivariate data from ASs, current BGP anomaly detection techniques are limited, and analysis is largely drawn from single monitoring points. Yet, the Internet is a complex phenomenon with many monitoring points and interactions, while the BGP itself is a nonlinear dynamical system that requires high-dimensional anomaly detection [12]. At best, most techniques seek to infer dynamics and information from single observables. Notwithstanding previously successful analyses of multivariate data and many features [11,12,13,14], such techniques are limited with respect to more advanced attacks. For example, an advanced BGP attack has been designed to avoid public collector infrastructure and target routing monitor blind spots to exploit single-observable approaches [8,15]. Extant techniques provide insufficient detection visibility, and advanced BGP detection techniques require capability at the multi-AS internetworked group level. This requires the adequate modeling of BGP speakers to investigate how groups of ASs are similar and differ from one another in terms of their interactions, as well as the dynamics of large groups of ASs.

Many techniques have been used to analyze BGP traffic from a single AS, or from single monitoring points, and inferences made from collectors; however, we model the BGP as a multidimensional, multi-observable system for the purposes of identifying candidate techniques that can capture and quantify the dynamics of multiple groups of distributed ASs for multi-viewpoint (MVP) anomaly detection. The ability to capture the group-level information and dynamics of ASs is key. This includes investigating our ability to detect BGP anomalies using multiple monitoring and vantage points and the effectiveness of using multiple peers in BGP collectors through identifying how the peers within collectors interact with each other, the group dynamics at play, and how peers in collectors may differ. In other words, the ability to capture, quantify, and use group-level AS information for group-level AS anomaly detection. Given that the BGP has been successfully shown to exhibit the characteristics of a nonlinear dynamical system [16], we posit that capturing and investigating the interactions and group dynamics among groups of ASs, be it a public or private collection and monitoring infrastructure, can facilitate advanced group-level anomaly detection techniques.

While both time series anomaly detection, generally, and BGP anomaly detection, specifically, have been previously surveyed [6,11,17], there exists no survey of the techniques capable of MVP BGP anomaly detection nor of the detection of advanced BGP attacks. Previous criteria for next-generation BGP anomaly detection are insufficient to detect some advanced BGP attacks, such as those that evade public collector monitors. This survey directly addresses security weaknesses in the BGP by identifying techniques that can enhance the resilience of BGP infrastructure against advanced attacks. This survey presents an evaluation of different techniques as evidence of their MVP capability and this next-generation anomaly detection requirement.Our contributions are that we:

- Establish the demand and conditions for an AS-group-level multi-viewpoint approach to the detection of advanced BGP attacks.
- Conduct a systematic survey of 178 unique anomaly detection techniques for the benefit of researchers.
- Identify possible MVP detection candidates for the detection of advanced BGP attacks that target route collector visibility limitations.
- Perform early exploratory analysis of, and report preliminary results from, experiments conducted using some of the identified candidates that have never before been applied to BGP anomaly detection.

The remainder of this paper is organized as follows. In Section 2, we summarize the functionality of the BGP. Section 3 describes the different types of BGP attacks. Section 4 and Section 5 frame the need for computationally efficient AS-group MVP anomaly detection. Section 6 evaluates all known attack categories based on their complexity and requirement for group-level MVP anomaly detection. Section 7 is a survey of 178 unique anomaly detection techniques, conducted for the purpose of identifying advanced MVP BGP anomaly detection candidates. Note that some techniques are identified and reside in more than one category. In Section 8, we briefly describe likely candidate approaches and evaluate two approaches in some preliminary detail. Section 9 discusses some future research opportunities and the paper concludes in Section 10.

## 2. Inter-Domain Routing and BGP

As the default inter-domain routing protocol for the Internet, the BGP has been described as a path-vector and distance-vector variant protocol [18,19]. Although internetworked routing domains (ASs) communicate using a shared protocol (BGP), they are autonomous entities administered by a single authority [20,21]. ASs are often large, complex groups of networks. Internetworked ASs are also not simply physically or geographically bound but rather formed by corporate, organizational, and political factors; topological-centric inferences about Internet operations can be flawed, with potentially important information on Internet interactions and dynamics lost in abstraction [3,4].

Every BGP message is structured with a consistent header that includes a marker, length, and type fields, totaling 19 octets in size. The marker field, which is 16 octets long and set entirely to 1, signals the beginning of a message. The length field, taking up 2 octets, specifies the overall message size, header included. The type field identifies the message’s category, which can be one of five types: OPEN, UPDATE, NOTIFICATION, KEEPALIVE, and ROUTE REFRESH, as specified in various RFCs (e.g., 1771, 4271, 2918, and RFC 7313 [22,23,24,25]). The initiation of a TCP session triggers the sending of an OPEN message, marking the start of the BGP message exchange necessary to reach the ESTABLISHED state. The termination and maintenance of sessions are communicated through NOTIFICATION and KEEPALIVE messages, respectively.

**Figure 1:**
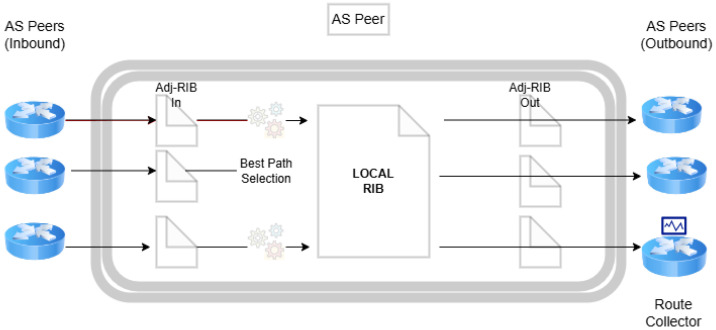
BGP-speaking router.

The storage of routes within a BGP peer is managed across three key databases, collectively referred to as the Routing Information Base (RIB): Adj-RIB-In, Adj-RIB-Out, and Loc-RIB (Figure 1). Adj-RIB-In is responsible for holding routes acquired from UPDATE messages from other peers, essentially reflecting the routes learned from neighboring peers that contribute to the path selection process. Conversely, Adj-RIB-Out archives routes that are disseminated to other peers, and Loc-RIB maintains the peer’s currently selected best routes, as determined by the Adj-RIB-In data and the peer’s internal path selection criteria. Modifications to routing information, such as announcements, withdrawals, or updates to existing attributes, are communicated following the RIB data exchange.

## 3. BGP Anomalies and Attacks

In this section, we provide a comprehensive overview of BGP anomalies and attacks, categorized based on their intent and impact. This section also discusses advanced BGP attacks that often evade detection by targeting blind spots in public BGP collectors. The discussion emphasizes the critical need for next-generation anomaly detection techniques capable of addressing these advanced threats by analyzing group-level AS interactions.

BGP anomalies and attacks have been studied extensively in the literature [6,10,11]. The severity of BGP incidents can range from the relatively innocuous (e.g., route flapping) through to destructive (e.g., BGP ‘hijacking’, rerouting, and ‘blackholing’), with both non-malicious or malicious intent [10,11]. BGP events (both malicious and non-malicious) have affected major Internet entities including Akamai, Apple, Amazon, Facebook, Google, Mastercard, and Microsoft [6,7,8]. In 2023, the Australian telecommunications company Optus experienced a BGP-related incident that impacted critical and emergency services [26,27,28].

A taxonomy of BGP anomalies has been previously constructed into the categories of direct intended anomalies, direct unintended anomalies, indirect anomalies, and outages (or link failures) [11]. Within each category, the authors further sub-classified BGP anomalies. The direct intended anomaly category represents the range of BGP hijacks currently known about (e.g., same-prefix, sub-prefix, stealth, and advanced BGP attacks). A network operator misconfiguration incident is a typical example of a direct unintended anomaly. Indirect anomalies are those affecting elements of Internet operations such as web servers. Examples of indirect events include significant cyber attacks (e.g., Nimda, Code Red II, and Slammer worm attacks) that affect ASs with intensified BGP activity and ultimately overload the Internet. For example, on the day preceding the Slammer worm incident, the average BGP announcement was 47 updates per prefix compared to the 4500 updates per prefix seen during the attack [29,30]. Outages are those stemming from natural disasters or energy system failures (e.g., Japanese Earthquake, Moscow Blackout).

BGP attacks have resulted in large volumes of the global Internet traffic being rerouted through state-owned entities, where numerous methods of cyber surveillance and retrospective forensics can be utilized. Nation-state-level actors can also manipulate the routing system to impose censorship [7,31]. This places at risk the many businesses, transactions, devices, and global matters of state present on this shared resource every day. There have been several BGP rerouting and interception attack incidents in recent years where nation-state-owned telcos have been observed to reroute traffic in events that impacted Akamai, Alfa-Bank, Amazon, Apple, EMF, Facebook, Fortis, Google, MasterCard, Microsoft, and Symantec, among others [6,31,32,33].

**Figure 2:**
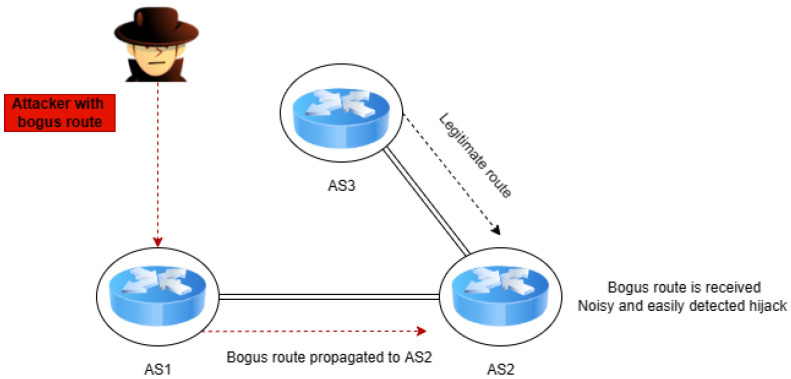
Noisy BGP hijack.

The nature and detectability of BGP attacks vary considerably [6,34]. While some are stealthy, evading detection with subtle manipulations, others are more conspicuous, creating significant ’noise’ in network activity (see Figure 2). These ‘noisy’ attacks often involve dramatic spikes in routing announcements or unusual patterns in AS path changes, making them more detectable but no less disruptive. Such attacks, due to their visible impact on routing data and network traffic flows, are particularly illustrative of the protocol’s vulnerabilities.

This section outlines the multifaceted nature of BGP attacks, categorizing them based on their objectives, techniques, tactics, and impacts, as outlined in the existing literature [6]. The discussion begins with data falsification-based attacks, where malicious entities manipulate or forge routing information, including the nuances of prefix and subprefix hijacking and AS path manipulation. These attacks disrupt data flows and compromise the integrity of data transmission [6,35].

Regarding the granularity of BGP hijacks, attacks such as same-prefix hijacking, subprefix hijacking, and AS path poisoning demonstrate the varied and increasingly sophisticated methods attackers use [6]. The subprefix hijack, for instance, exploits the BGP’s trust model by announcing a more specific prefix, thereby rerouting traffic. Path poisoning attacks leverage the BGP’s loop prevention mechanism, selectively inhibiting route propagation by including specific ASNs in the path. While such attacks are detectable through certain measures, the emergence of more stealthy and surgical interception techniques, including the strategic use of BGP communities’ attributes, presents ongoing challenges in ensuring secure and reliable routing [34,36,37].

Protocol manipulation-based BGP attacks are those where attackers exploit the BGP’s decision-making mechanisms [6]. Techniques such as altering the Multi-Exit Discriminator (MED) or exploiting timers such as Route Flap Damping (RFD) and the Minimum Route Advertisement Interval (MRAI) allow adversaries to influence route selection and destabilize networks [37,38]. Data misuse attacks are explained through attack methods such as denial of service (DoS) and route leaks [6,39,40]. Lastly, recent advanced and evasive BGP attacks have been described in the literature, where attackers equipped with in-depth knowledge of BGP infrastructure execute sophisticated and stealthy operations, often evading detection and exploiting the public collector and monitor’s infrastructure and their limitations [15].

### 3.1. Prefix Attacks

Previous research has shown that up to 72% of domains are vulnerable to the most basic of BGP subprefix hijacks and up to 70% are vulnerable to same-prefix attacks [41,42]. Research has also shown the ease with which bogus certificates can be obtained from the top five Certificate Authorities (CAs), with all being susceptible to standard BGP hijack attacks. Following these validated attacks, some CAs began implementing mitigation, though highly targeted surgical BGP attacks using stealth remain a threat [41,43].

**Figure 3:**
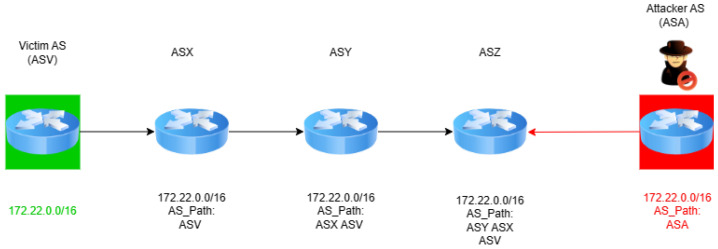
Prefix hijack.

Traditional prefix hijacks involve the unauthorized announcement of IP prefixes, effectively attracting traffic intended for the legitimate prefix owner towards a malicious AS. The simplicity yet effectiveness of traditional prefix hijacks have been demonstrated in numerous instances, causing widespread disruption and posing significant challenges in their mitigation [6]. Prefix hijacking involves an attacker AS (ASA), such as the one in Figure 3), intentionally announcing IP prefixes that it does not own, thereby misleading traffic through unauthorized paths. As depicted in Figure 3, ASA falsely claims ownership of the IP prefix 172.22.0.0/16, which actually belongs to ASV (the victim).

This unauthorized announcement is strategically made to ASX, ASY, and particularly ASZ, positioning ASA as a seemingly legitimate intermediary for traffic destined for ASV. By doing so, ASA attempts to force ASZ, a critical juncture in the routing path, to adopt these fake routes, effectively diverting the traffic through ASA. This diversion disrupts the intended routing path and opens up avenues for additional malicious activity, as ASA gains undue control over the traffic flow meant for ASV. The impact of such attacks is multifold, leading to potential traffic interception, data theft, and denial of service, thereby posing a significant threat to the confidentiality, integrity, and availability of data traversing the Internet.

In addition, prepended prefix hijacks introduce an additional layer of complexity, involving the manipulation of an AS path prepending to influence BGP route selection. By artificially inflating the AS path length, attackers can subtly influence traffic flows, redirecting them through malicious or unintended paths, thereby enabling the analysis, interception, or even manipulation of the traffic. Real-world instances of prepended prefix hijacks have highlighted the challenges in detecting and mitigating them, given their subtle and often inconspicuous nature, which may not immediately disrupt traffic flows or raise alarms.

Both traditional and prepended prefix hijacks highlight a spectrum of challenges and considerations in their detection and mitigation. While both hijack types exploit the BGP’s inherent trust and lack of authentication, their tactics, stealth, and impact can vary significantly, thereby necessitating a nuanced approach to their mitigation. The development and implementation of countermeasures, such as path validation, prefix filtering, and cryptographic validation mechanisms, have been explored to various degrees, yet the decentralized and trust-based architecture of the BGP continues to pose persistent challenges in ensuring robust, secure inter-domain routing. In summary, prefix hijacks, in their various forms, underscore the imperative for enhanced security mechanisms within the BGP.

### 3.2. Subprefix Attacks

Subprefix hijacking, a nuanced form of the BGP routing attack, has garnered attention for its ability to surreptitiously divert Internet traffic through unauthorized paths, thereby enabling a range of malicious activities, including traffic interception, surveillance, and potentially data manipulation. In a traditional subprefix hijack, a malicious AS intentionally announces a more specific IP prefix than that of the legitimate owner, effectively luring traffic destined for the legitimate prefix towards the attacker. Given the BGP’s preference for more specific prefixes in its routing decisions, routers across the Internet are duped into redirecting traffic through the malicious AS, often unbeknownst to both the legitimate prefix owner and the unsuspecting users whose data are now at the mercy of the attacker. The implications of traditional subprefix hijacks can be devastating, enabling attackers to eavesdrop on data, perform adversary-in-the-middle attacks, or even blackhole traffic, thereby disrupting communication and potentially causing significant operational and reputational damage.

**Figure 4:**
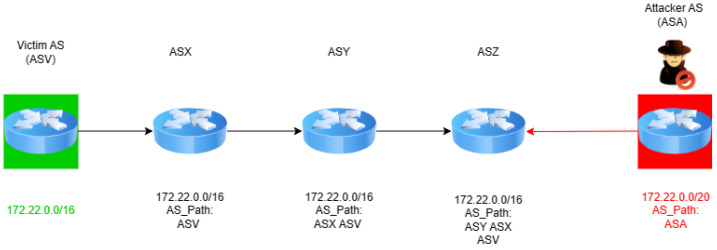
Subprefix hijack.

In the scenario illustrated in Figure 4, ASA (the attacker) perpetrates a subprefix hijack by falsely announcing ownership of a more specific IP prefix, 172.22.0.0/20, which is a subset of ASV’s (the victim’s) legitimately owned broader prefix, 172.22.0.0/16. This strategic announcement by ASA leverages the BGP’s longest prefix match rule, misleading ASX, ASY, and ASZ into routing traffic that was intended for ASV through ASA instead. ASA’s manipulation of the BGP’s routing decisions not only intercepts the traffic destined for ASV but also potentially subjects it to unauthorized surveillance or manipulation. By exploiting this fundamental aspect of BGP routing, ASA effectively diverts Internet traffic, undermining the integrity and confidentiality of data transmissions to ASV.

Prepended subprefix hijacking not only announces a more specific prefix but also manipulates the AS path through prepending, artificially inflating the path length in a bid to influence routing decisions subtly. While the BGP inherently prefers shorter AS paths, strategic prepending allows the attacker to craftily manipulate traffic flows, enabling them to target specific ASs or regions without causing widespread disruption or detection. The subtlety of prepended subprefix hijacks poses significant challenges in their detection and mitigation, as their malicious routes may not universally propagate and may only impact specific, targeted portions of the Internet.

Both traditional and prepended subprefix hijacks exploit the BGP’s preference for more specific prefixes and its trust-based operational paradigm, yet they differ in their execution, stealth, and potential detectability. Mitigating such attacks necessitates a multifaceted approach, involving the deployment of prefix filtering, Route Origin Authorization (ROA), and the adoption of the Resource Public Key Infrastructure (RPKI) to validate the authenticity of BGP announcements. However, the decentralized nature of the BGP and the varied adoption of security practices across ASs worldwide continue to pose hurdles in universally securing the BGP against subprefix hijacks. Improved anomaly detection technologies and capabilities are required.

### 3.3. AS Path Forgery

AS path forgery strategically manipulates the AS path attribute in BGP announcements to mislead routers and divert Internet traffic through unintended paths. This attack category, while potentially not as overtly disruptive as prefix or subprefix hijacking, carries its own set of unique challenges and threats to the stability and security of inter-domain routing.

In a typical AS path forgery attack, the malicious actor announces IP prefixes with a manipulated AS path, altering the sequence of ASs that the announcement has traversed. This could involve injecting additional AS numbers, reordering existing ASs, or even spoofing the origin AS, thereby presenting a falsified path to receiving routers. The receiving routers, trusting the received BGP announcement, update their routing tables accordingly, inadvertently directing traffic through the attacker’s AS, or another AS of the attacker’s choosing.

**Figure 5:**
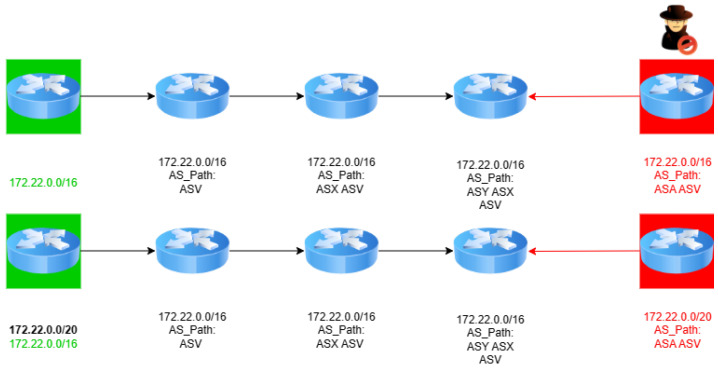
AS path forgery hijacks.

In the first scenario, depicted in the upper portion of Figure 5, we can observe the one-hop prefix hijack, a sophisticated instance of AS path forgery. Here, ASA (the attacker) crafts a BGP announcement for the IP prefix 172.22.0.0/16, incorporating a fabricated AS path that falsely indicates a direct connection to ASV (the victim). This manipulated announcement misleads ASX, ASY, and ASZ into routing traffic intended for ASV through ASA under the guise of offering a more direct or efficient path (ASA, ASV). Such a maneuver not only disrupts the intended flow of data but also exposes it to unauthorized interception or manipulation by ASA, thereby compromising the security and integrity of data directed towards ASV.

The second scenario, illustrated in the lower portion of Figure 5, describes a one-hop subprefix hijack. In this refined attack, ASA announces the route of a more specific IP prefix, 172.22.0.0/20, carving out a segment from ASV’s broader 172.22.0.0/16 network. The announcement includes a forged AS path that suggests a non-existent direct link to ASV, misleading other ASs to route a portion of the traffic through ASA. This strategy exploits the BGP’s longest prefix match rule to siphon off traffic meant for ASV, allowing ASA to intercept, analyze, or manipulate the data in transit.

AS path poisoning is another example within the AS path forgery category of attack that involves the intentional insertion of AS numbers into the AS path attribute of a BGP announcement to influence the propagation of the route and potentially prevent certain ASs from receiving it. By “poisoning” the AS path with specific AS numbers, the attacker can manipulate the BGP’s loop-prevention mechanism, which discards routes containing its own AS number in their AS path, thereby controlling the propagation of the malicious BGP announcement. This can be utilized to perform targeted attacks, create routing asymmetry, or to avoid detection by specific ASs or monitoring systems.

Some researchers have further defined this category of attacks into Type-1 to Type-5 attacks:

- Type-1 Attack: The attacker (ASA) strictly claims to be a neighbor of the victim (ASV) by announcing a forged AS path A, V. This is a direct assertion of false adjacency and is a clear example of AS path forgery.
- Type-2 to Type-5 Attacks: These involve extending the forged AS path, claiming to be progressively further from the ASV in terms of AS hops. The longer the forged path, the stealthier, but potentially less impactful, the attack becomes in terms of traffic redirection.

While the prepended (sub)prefix attacks described in previous sections influence route selection, AS path forgery directly deceives routers about the path’s legitimacy or origin. AS path poisoning intentionally makes a route unattractive or unacceptable to specific ASs. In summary, they define the forging of AS path in the BGP announcement to claim different levels of proximity to the victim.

### 3.4. Interception Attacks

Interception attacks, particularly those executed as adversary-in-the-middle (AitM) operations within the BGP ecosystem, represent a sophisticated evolution of prefix hijacking techniques. Unlike traditional hijacks that merely redirect traffic, interception attacks are characterized by their ability to both divert and subsequently forward traffic, ensuring that communications reach their intended destinations, albeit via the attacker’s network. This dual action allows the attacker to remain undetected, preserving the connectivity and functionality of the network while clandestinely monitoring or manipulating data.

At the heart of an interception attack is an attacker’s ability to insert themselves as a clandestine intermediary within the communication path between a source and its intended destination. By exploiting vulnerabilities in BGP routing, attackers announce routes that not only attract traffic towards their systems but also cleverly reroute it back to the legitimate path after inspection or alteration. This sophisticated strategy involves announcing IP prefixes—either exact matches or more specific subprefixes not owned by the attacker—to mislead routers into sending traffic through the attacker’s network.

The execution of an interception attack typically involves two critical phases: the initial diversion of traffic through the attacker’s network and the subsequent forwarding of this traffic back to its original path. This process requires a nuanced understanding of BGP routing mechanisms and the ability to manipulate route announcements in a way that remains invisible to both the source and destination ASs. By maintaining the appearance of normalcy, the attacker can intercept, inspect, and potentially alter data packets without raising alarms, making interception attacks particularly insidious and difficult to detect. The DEFCON attack is another well-known experiment that demonstrates interception attacks on the Internet.

**Figure 6:**
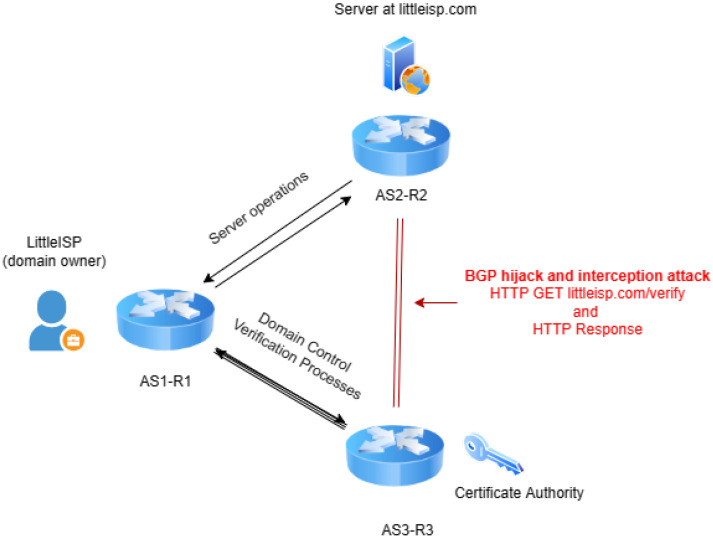
BGP hijacks and interceptions to compromise CAs.

An example of the application of an interception attack might involve compromising the integrity of CAs (illustrated in Figure 6). CAs are pivotal in Internet security, issuing digital certificates that verify the identity of websites and ensure encrypted connections. However, by manipulating BGP routes, attackers can intercept the validation checks performed by CAs [36,41,43]. This interception enables the attacker to fraudulently obtain CAs for domains they do not own, facilitating a range of malicious activities. There have also been a number of suspected geopolitical BGP interception incidents in recent years that include Internet traffic being compromised and rerouted through state-owned telecommunications companies and ISPs [6,31,32].

### 3.5. Replay and Suppression Attacks

Replay and suppression attacks in the realm of BGP security present a nuanced challenge, intertwining the stability and reliability of inter-domain routing with the malicious intent of adversaries seeking to exploit the BGP’s inherent trust and lack of authentication. The mechanics of these attacks are rooted in the manipulation of BGP withdrawal messages, which are pivotal in maintaining the integrity and optimality of routing paths within the network.

In a replay attack, an adversary retransmits a previously announced route, potentially resurrecting an outdated or invalid path into the routing tables of BGP speakers. This is not merely a reiteration of old data but a strategic move to introduce instability within the network, causing routers to reevaluate and potentially switch to these suboptimal paths. The implications of such an attack can be widespread, affecting not only the direct recipients of the malicious announcements but also indirectly impacting ASs that might select the revived path as a new optimal route. The cascading effect of this can lead to traffic being routed through unintended paths, which might be longer, less secure, or even controlled by the adversary, thereby enabling further malicious activities such as eavesdropping or data interception.

Comparatively, a suppression attack is characterized by the intentional non-propagation or delay of BGP withdrawal messages. When a route becomes invalid or an alternative, more optimal path is identified, a withdrawal message is issued to inform neighboring ASs of the change, prompting them to update their routing tables. By suppressing these messages, the attacker ensures that routers continue to utilize an outdated or suboptimal path, thereby exerting a level of control over the flow of data within the network. This could facilitate various malicious endeavors, such as traffic analysis and data interception, or simply cause degradation in network performance by forcing data to traverse longer or less efficient paths.

An insidious element of replay and suppression attacks is observed in their ability to manipulate the control plane without causing immediate or overt disruptions, thereby allowing the attacker to sustain their activities over prolonged periods without detection [6,44]. This subtle manipulation of the routing tables across a network can be exploited in various ways, such as facilitating other types of attacks, creating routing inefficiencies, or simply causing instability within the network.

### 3.6. Collusion Attacks

Unlike other attacks, which may originate from a single AS, collusion attacks involve the cooperative malicious behavior of two or more non-neighboring ASs [6,45]. These ASs create a virtual tunnel between them, establishing a BGP session through which they can exchange and propagate forged routing information without causing conspicuous routing conflicts.

In a typical collusion attack scenario, the malicious ASs agree to accept and propagate each other’s illegitimate BGP announcements [6,45]. This cooperative malicious activity can facilitate a range of other attacks, such as prefix hijacking or AS path spoofing, by providing a mechanism through which malicious BGP announcements can be injected into the global BGP system and propagated to other, non-malicious ASs. The colluding ASs can, for instance, agree to propagate BGP announcements that contain IP prefixes that neither AS is authorized to advertise, or that contain forged AS path attributes, thereby manipulating the path of Internet traffic on a global scale.

The threat posed by collusion attacks is due to the inherent trust that underpins the BGP. ASs generally trust the BGP announcements received from their peers, and this trust can be exploited by colluding ASs to inject malicious routing updates into the global BGP system [6,45]. This can facilitate various malicious activities, such as traffic interceptions, traffic analyses, or denials of service, by manipulating the path of Internet traffic to traverse malicious ASs or to avoid legitimate ones.

### 3.7. MED Modification Attacks

MED modification attacks focus on manipulating the BGP attribute known as the Multi-Exit Discriminator (MED), which is utilized by ASs to convey to their neighbors a more preferred path for incoming traffic [37,38]. The MED is a crucial attribute in influencing route selection, especially in scenarios where multiple paths exist between two ASs. By maliciously modifying the MED values in BGP announcements, an attacker can influence the path selection process, causing traffic to be routed through specific, potentially malicious, paths. This could be leveraged to facilitate traffic interception, create network congestion, or simply degrade network performance by forcing traffic through suboptimal paths. The subtlety of MED modification attacks lies in their ability to manipulate routing decisions without violating the BGP’s path selection rules or causing overt routing conflicts.

**Figure 7:**
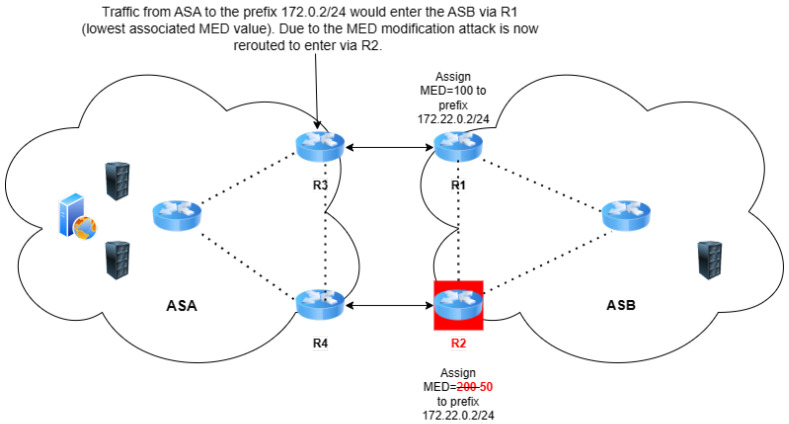
MED modification.

A MED modification attack is illustrated in Figure 7, within the context of a Tier 3 ISP. ASB has two eBGP sessions toward the same upstream provider ASA. Initially, ASB advertises its prefix 172.22.0.2/24 on both eBGP sessions—one ending on R3 with a MED value of 100, and the other on R4 with a MED value of 200. Initially, ASB advertises the prefix 172.22.0.2/24 to ASA through two eBGP sessions, with different MED values assigned to each advertisement for traffic engineering purposes. In a malicious act, the MED value for the advertisement from R2 (ASB) to R4 (ASA) is altered from 200 to 50, making this path artificially more attractive to ASA. In contrast to an attack such as AS path forgery, which falsifies the sequence of ASs to manipulate the perceived path of a route, a MED modification attack influences route selection by altering the MED value without changing the AS path itself.

### 3.8. RFD/MRAI Timer Exploitation

Exploiting the RFD and MRAI timers introduces another layer of protocol manipulation, wherein the attacker seeks to exploit the BGP’s mechanisms for mitigating route flapping and controlling the frequency of BGP announcements. Route Flap Damping (RFD) is designed to stabilize the BGP by suppressing routes that flap frequently, while the Minimum Route Advertisement Interval (MRAI) timer controls the minimum time interval between consecutive BGP updates, aiming to reduce the load on BGP routers and the number of BGP update messages in the network. By strategically manipulating BGP announcements to exploit these mechanisms, an attacker could potentially cause legitimate routes to be suppressed, create routing instability, or manipulate the propagation of BGP updates across the Internet. For instance, by intentionally flapping a route, an attacker could trigger RFD and cause the route to be suppressed, thereby influencing routing decisions and potentially enabling other types of attacks.

### 3.9. Denial of Service (DoS)

Denial of service (DoS) attacks, in the context of the BGP, involve the malicious manipulation of routing tables and paths to render a network, or parts of it, inaccessible. BGP DoS attacks can be particularly insidious as they can disrupt the flow of data across multiple networks, not just the immediate target. In a BGP DoS attack, the attacker may manipulate BGP announcements to either drop traffic destined for a particular prefix or to create routing loops. This could involve announcing IP prefixes that do not belong to the attacker, causing traffic to be misrouted, or intentionally withdrawing legitimate BGP announcements, causing network outages.

The impact of a BGP DoS attack can be widespread, causing network outages, degraded service quality, and potentially leading to cascading failures across interconnected networks. The misrouting or dropping of traffic can disrupt critical services and communications, with potential socio-economic consequences. The mitigation of BGP DoS attacks often involves implementing prefix filtering, ensuring that only legitimate BGP announcements are accepted. Utilizing the Resource Public Key Infrastructure (RPKI) to validate the authenticity of BGP announcements and employing measures for path validation are examples of extant mitigation measures.

### 3.10. Monitor-Evasive Attacks

Increasingly smarter attacks have been designed to avoid public BGP collector and monitor infrastructures; these are effectively deployed as ’monitor-aware’ attacks, as has been described in [15]. BGP policies or communities can also be manipulated to assist in advanced attacks (e.g., MED modification, Local Preference manipulation, or BGP community tagging and engineering). These attacks leverage knowledge of the global BGP policy landscape and specific vulnerabilities in the route selection process to ensure that the malicious path, while passing through an AS, does not trigger any alarms or become preferable to the paths monitored by public collector infrastructure [15].

**Figure 8:**
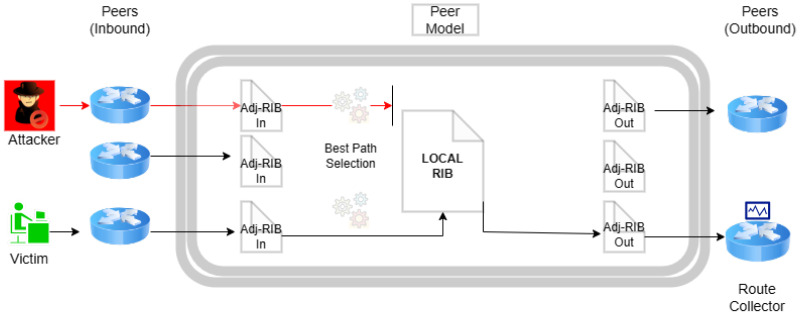
Attack was neither stored nor detected.

Thus, monitor-evasive attacks are those those that can evade public route collection infrastructure by limiting the propagation of their attack, strategically identifying ASs in the announced path, increasing the announced AS path length, and exporting the attack to the chosen network victims [15]. In other words, advanced BGP attacks can avoid detection by public Internet infrastructure in two ways; either the peer has no visibility of the attack, as it did not propagate via any neighboring ASs, or the attack has propagated to the peer but the hijack was not stored in the Loc-RIB, therefore the victim’s path was propagated to the collector and no anomaly was detected. For example, consider the model of a BGP speaker that is illustrated in Figure 1; the hijacked announcement will not be stored or propagated to the collector (Figure 8).

Advanced attacks can partition Internet regions into ASs that will preference the attacker’s AS path and those that will preference a victim’s AS path for the purposes of evading public route collection infrastructure [15]. The partitioning of ASs into groups for evasive BGP attacks means that any detection scheme must capture high-dimensional group-level interactions, dynamics, and information. There are very few studies that have investigated this type of attack, but a rigorous description and analysis of the taxonomy and function of monitor-evasive attacks can be found in the literature [15,46]. The attacker ensures that its bogus announcements bypass the public BGP route collectors. Studies show that current collection strategies do not provide comprehensive visibility of the global routing system and are hence vulnerable to these attacks [15,47].

All existing BGP anomaly detection schemes are designed from single observables and almost entirely rely on public collector infrastructure. Monitor-evasive attacks have been designed to avoid public collector infrastructure and exploit single-observable approaches, as they provide limited, narrow-view detection visibility. Research suggests that future work should investigate the strategic placement of monitors and the novel optimization of Internet routing measurements [15,47,48]. However, historical research conducted on peer monitor selection and collector infrastructure, while significant, has largely been topologically based [49,50]. Topological methods do not capture the high-dimensional dynamics of the communication system (BGP) nor the interactions and information of ASs at the group level. The hypothesis in this regard is straightforward; can a technique that captures the group interactions, dynamics, and information of ASs provide more information to detect advanced BGP attacks that currently avoid known techniques?

In addition to the reliance on route collection infrastructure as an already identified reason for monitor-evasive attacks defeating extant anomaly detection approaches, we also hypothesize that all known detection schemes are largely focused on, and limited to, single observables that can be used to analyze an AS and BGP activity from single monitoring points. As such, current detection schemes have no proven nor proposed capability to encapsulate, quantify, and utilize group-level interactions, dynamics, and information across multiple ASs (e.g., AS monitors and collector infrastructures) for the purposes of the MVP anomaly detection of advanced BGP attacks. Hence, there is a requirement for next-generation advanced BGP anomaly detection techniques that can quantify the dynamics of large groups of ASs and provide high-dimensional anomaly detection. We will next further outline this critical criterion for next-generation BGP anomaly detection.

Current BGP anomaly detection techniques face limitations due to the fact that their analysis is drawn from single monitoring points, even when multiple single monitoring points are analyzed. While existing approaches to BGP anomaly detection techniques do not meet the MVP requirement for next-generation detection, previous research on BGP-powered attacks on Certification Authorities (CA), such as those previously outlined in Section 3 (Figure 6), has shown that deploying more vantage points can be successful for mitigation. The findings illustrated that defense from a single observable resulted in the victim domain mitigating 17% of attacks; with the addition of more vantage points, the victim was resilient to 85% of attacks [41,42].

## 4. Why the Need for Low-Parameter Computationally Efficient Techniques?

This section highlights the necessity of low-parameter and computationally efficient anomaly detection techniques in BGP-speaking routers. Due to the limited computational resources, such as CPU power, memory, and energy, available in BGP routers, these devices must prioritize handling routing updates and BGP messages. Complex, parameter-heavy algorithms for anomaly detection introduce processing delays and scalability challenges, compromising the router’s core functions and network performance. Lightweight and fast anomaly detection techniques are necessary to ensure timely threat identification and resilience without overburdening network infrastructure. This section emphasizes how reducing the number of parameters used in detection techniques is crucial for maintaining both speed and scalability in large-scale BGP networks.

BGP-speaking routers are constrained by limited computational resources, including CPU power, memory, and energy [51,52,53]. These resources are primarily dedicated to handling routing table updates and processing BGP messages. Therefore, any additional functionality, such as anomaly detection schemes, must be implemented in a resource-efficient manner to avoid compromising the router’s primary functions [53,54,55]. The high-speed and large-scale nature of BGP-speaking networks demands fast, lightweight, and computationally efficient anomaly detection techniques to ensure timely threat identification and network resilience [56,57].

**Figure 9:**
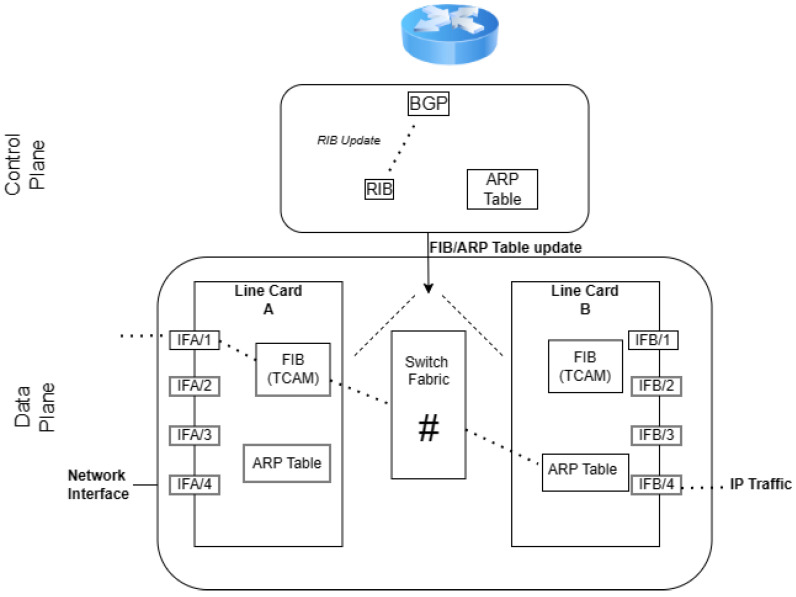
BGP-speaking router control and data planes.

The environment of BGP routing requires rapid decisions to ensure efficient data packet forwarding [53,55]. Introducing complex algorithms or models for anomaly detection can lead to processing delays, affecting network performance and stability. Both the Routing Information Base (RIB) and the Forwarding Information Base (FIB) (see Figure 9) require rapid updates and lookups to adapt to changes in network topology and routing policies. Complex algorithms can extend these processing times, causing delays in routing decisions [54,58].

In environments where latency in routing decisions is critical, heavyweight computational or parameter-heavy models can increase processing latency, undermining the timely delivery of network traffic [56]. Additionally, BGP routers operate in large-scale networks where scalability is essential. Lightweight and computationally efficient programs are preferred to ensure scalability across numerous routers without overburdening the network infrastructure [54]. Heavy computational tasks can also significantly impact energy consumption.

Efficient anomaly detection techniques for BGP routers should avoid the imposition of numerous features, parameters, domain-specific tuning, and extensive training, as these can counteract the goals of speed, lightweight operation, and computational efficiency. The pressures imposed by numerous features and parameters can impact their computational complexity, resource intensiveness, latency, and response time.

Introducing a multitude of features and parameters into anomaly detection algorithms elevates the computational complexity of BGP routers. Each additional feature or parameter adds to the processing overhead, potentially leading to performance bottlenecks and degraded throughput. This increased complexity runs counter to the objective of lightweight and nimble operation required in BGP networks [54,56].

Domain-specific tuning and training can intensify resource demands on a BGP router. Training algorithms to recognize anomalies within the BGP-speaking routing system requires substantial computational resources and extensive datasets. Furthermore, the iterative nature of training processes demands ongoing maintenance and updates, further straining the limited resources available to BGP routers.

The accumulation of features, parameters, and domain-specific tuning imparts latency and delays to the anomaly detection process [52,56]. BGP routers must rapidly analyze incoming data streams to promptly identify and mitigate potential anomalies. However, the computations associated with complex anomaly detection algorithms can introduce latency into the decision-making pipeline, potentially impeding critical tasks such as route convergence and traffic engineering.

Anomaly detection techniques laden with features and parameters face scalability challenges in large-scale BGP networks. As the network’s size and complexity increase, the computational demands placed on anomaly detection systems escalate proportionally [54,57,59]. Scaling traditional anomaly detection approaches to accommodate expansive BGP infrastructures requires substantial investments in hardware resources and computational infrastructure.

In essence, techniques reliant on numerous features, parameters, domain-specific tuning, and training heighten computational complexity, resource intensiveness, latency, response time, and scalability challenges. These factors collectively undermine the objectives of fast, lightweight, and computationally efficient anomaly detection in BGP routers. This highlights the need for computationally efficient, low-parameter approaches that prioritize speed, agility, and resource optimization.

## 5. Why the Need for a Group-Level AS Anomaly Detection Technique?

This section highlights the importance of group-level anomaly detection techniques in capturing the collective information and dynamics of interconnected ASs within a BGP system. Understanding multi-viewpoint group dynamics will be important for detecting advanced BGP attacks, which often exploit single-observable detection systems. Current BGP anomaly detection methods are limited by their focus on individual AS observables. To effectively detect sophisticated attacks that avoid public routing infrastructure, next-generation techniques must analyze the interactions and dynamics of multiple ASs simultaneously.

From the coordinated movements of flocks of birds and schools of fish to the collective behaviors of human groups, understanding group-level information and behaviors is an active area of research [60]. Ongoing research endeavors aim to develop holistic approaches that consider the interactions and interdependencies among entities at the group level, enabling a deeper understanding of complex system behaviors. The effective detection of advanced attacks across the vast BGP-speaking Internet also necessitates capturing the collective dynamics of interconnected ASs. Parallels can be drawn from natural phenomena to complex, dynamic, and networked computer systems, such as the inter-domain-routed Internet. In each case, the challenge lies in deciphering the intricate dynamics that emerge from interactions among individual entities within the collective.

Insight into collective behaviors extends beyond natural phenomena. In quantum error correction and aperiodic tilings, researchers have shown that grasping the global properties of complex systems requires analyzing collective dynamics, rather than focusing solely on local details [61]. For example, examining a small portion of an aperiodic tiling, such as a Penrose tiling (or Ammann–Beenker tiling), presents a challenge in the inference of the overall structure or properties of the entire tiling from that small portion alone. The local arrangement of the tiles does not provide sufficient information to deduce their global structure due to the complex and non-repetitive nature of aperiodic tilings [61]. Similarly, in Quantum Error-Correcting Codes (QECCs), researchers have identified that observing or interacting with only a small part of the quantum system (such as a qubit or a few qubits) may not yield a comprehensive insight into the entire encoded quantum information or the overall state of the system [61]. The challenge arises because the encoded quantum information is spread across the entire system in a highly entangled manner, and local measurements do not reveal the full extent of the encoded information.

Consider a busy city mall, where each individual represents an AS, and their interactions mirror the exchanges between ASs (e.g., BGP messages). A performer begins an impromptu show, representing a BGP incident (e.g., a leak or hijack). The reactions among the individuals are varied and complex: some stop to watch, others move away, a few start recording the event, some call others over, and a few might even join the performance. Individuals experience physiological changes in response to the event, such as increased heart rates, pupil dilation, and changes in galvanic skin response.

These diverse reactions can be likened to the different BGP features extracted from incident data and used for BGP anomaly detection, such as the number of announcements, withdrawals, and average AS path length. Analyzing these reactions using standard techniques that focus on single AS observables—even if these individual reactions are correlated—offers a fragmented view of the overall situation. It is comparable to trying to understand the full impact of the performance by looking at only one group of individuals. What are needed are anomaly detection techniques that can capture the collective dynamics of the mall during the incident (a BGP attack)—how different groups of individuals (ASs) are affected and respond in various ways—reflecting the dynamics of a large, interconnected system.

For instance, the way some individuals quickly organize alternative plans might influence others to join them, or the calm demeanor of a seasoned traveler might reassure those around them. These collective dynamics provide a richer, more nuanced understanding of the situation, akin to completing a puzzle and understanding how each piece contributes to the whole picture. In the context of BGP, techniques capable of analyzing the group dynamics of ASs during an incident may offer insights that are more detailed and valuable than those obtained from analyzing single ASs or correlating multiple single ASs. This requires us to have the ability to observe and analyze multiple ASs simultaneously, capturing the intricate interplay among them.

A multi-observational, grouped AS approach mirrors the reality of BGP operations within the complex, distributed system of the Internet, where ASs are interconnected in a dynamic and distributed manner. The importance of addressing the visibility limitations of current approaches is only heightened with recent descriptions of ’smart’ BGP attacks that can avoid public routing infrastructure, specifically targeting visibility limitations and vulnerabilities [15]. A promising direction for enhancing the exploration of group-level AS BGP anomaly detection also involves leveraging recent advancements in strategic VP methodologies for optimized data collection (see [48]).

A range of approaches exist for the detection of BGP anomalies. An earlier review of BGP anomaly detection techniques categorized detection approaches into machine learning, reachability-based approaches, statistical pattern recognition, time series analysis, and validation studies based on historical BGP data [11]. While [11] further categorized each study by technique, type of anomaly detected, and whether the source of the anomaly could be identified, several attacks demonstrate the requirement for additional next-generation BGP anomaly detection criteria. We add group-level AS MVP as a requirement for next-generation anomaly detection, given that sophisticated BGP attacks have continued to evolve [15,36].

## 6. Attacks that Require Advanced Detection

This section evaluates various BGP attacks. It categorizes attacks into groups such as prefix hijacking, subprefix hijacking, AS path forgery, AS path poisoning, interception attacks, and others, while assessing the complexity and need for group-level AS and MVP analyses. This section discusses how certain attacks, due to their distributed and stealthy nature, require advanced detection techniques that leverage multiple viewpoints across the Internet. A detailed evaluation table is provided to classify these attacks based on their potential to benefit from advanced detection methods, including whether they involve complex interactions, stealthiness, or evasion tactics. The analysis emphasizes the need for sophisticated detection approaches capable of understanding grouped AS dynamics for early the identification of such advanced attacks.

We evaluate all known categories of BGP attacks based on previous research [6], with some recent advanced attacks included [15]. Focus is on the attack characteristics that might benefit from an advanced detection technique, leveraging group-level AS and multiple-vantage-point analysis and detection. The following is a summary of the inclusion and exclusion criteria used to evaluate the suitability of BGP attacks for advanced anomaly detection techniques that leverage group-level AS analyses and MVP observations.

- **Inclusion Criteria:**
  − MVP Detection: Attacks detectable earlier through the correlation of data from multiple network viewpoints, revealing inconsistencies in BGP announcements (e.g., attack surfaces and temporal elements).
  − Collaborative or Distributed Nature: Attacks involving collusion between ASs, requiring group-level analysis to detect coordinated malicious activities.
  − Complex AS Interactions: Attacks involving intricate routing dynamics across multiple ASs that require an understanding of AS relationships for detection.
  − Sophisticated BGP Manipulation: Advanced attacks where the manipulation of routing information is subtle and requires a multi-AS viewpoint analysis to detect.
  − Stealthy/Evasive Techniques: Attacks designed to evade conventional monitoring, including those that selectively announce or alter AS path attributes to bypass public route collectors.
- **Exclusion Criteria**:
  − *Simple Attacks*: Direct attacks such as basic prefix hijacking, which are easily detectable without sophisticated multi-point analysis.
  − *Non-BGP Attacks*: Attacks relying on vulnerabilities outside the BGP, such as non-protocol-layer attacks.
  − *Non-Strategic Impact*: Attacks that do not influence BGP routing decisions strategically or involve complex AS-level interactions.

Table 1 details the criteria that were used to evaluate advanced BGP attacks. In Table 2, each of the BGP attacks is evaluated based on these criteria, with an assessment made of their comparative attack complexity.

### 6.1. Prefix Hijacking

Traditional prefix hijacking is not characterized by stealthiness or evasion tactics designed to bypass detection mechanisms. It is relatively straightforward and detectable by several extant monitoring systems that look for anomalies in prefix ownership. It is a direct, simple attack that does not inherently involve the sophisticated manipulation of routing information, eitehr collaborative or distributed nature, or stealthiness and evasion tactics that would necessarily require a group-level analysis for detection. While it does affect routing across multiple ASs, the nature of the attack and its detection do not require the nuanced understanding of AS interactions and dynamics envisioned for more complex attacks.

While prefix hijacking is typically an attack executed by a single AS, the impact of the attack is distributed across the Internet, affecting data paths across multiple ASs. However, the attack itself does not involve collusion between these ASs. Therefore, this criterion may not strongly apply to prefix hijacking.

However, prefix hijacking, by its nature, can significantly benefit from early detection through multi-vantage point analysis. Discrepancies in BGP announcements, such as an unauthorized AS announcing a prefix it does not own, can be more readily identified when data from multiple points in the Internet are analyzed. This comprehensive view allows for the detection of anomalies that might not be visible from a single vantage point, making prefix hijacking a candidate for inclusion based on this criterion.

### 6.2. Subprefix

Subprefix hijacking might benefit from detection methods that leverage data from multiple vantage points. This is because the attack involves announcing a more specific prefix than the legitimate owner, which can be harder to detect across different points in the Internet. The ability to correlate discrepancies in BGP announcements from multiple sources can lead to the earlier detection of such hijacks, making subprefix hijacking a strong candidate for inclusion based on this criterion.

Subprefix hijacking is typically executed by a single AS without the need for collusion between ASs. Its distributed impact of the attack across the Internet does not inherently involve collaborative malicious activities between multiple ASs. Therefore, this criterion may not strongly apply to subprefix hijacking. The nature of subprefix hijacking, where a more specific route is announced to attract traffic, can involve complex interactions across multiple ASs. The attack exploits the BGP’s preference for more specific prefixes, affecting routing decisions and potentially causing widespread disruption. Understanding these interactions is crucial for detection, aligning well with this inclusion criterion.

Subprefix hijacking can involve a nuanced manipulation of routing information, exploiting the granularity of prefix announcements to reroute traffic subtly. This level of manipulation requires a detailed understanding of the BGP’s operational principles. In terms of stealthiness and evasive techniques, the specificity of subprefix hijacking can make it more stealthy compared to broad prefix hijacks, as it might not immediately disrupt traffic flows or raise alarms.

### 6.3. AS Path Forgery

AS path forgery is neither direct nor simple, as it requires careful planning and execution to be successful and to remain undetected. AS path forgery, by altering the AS path attribute in BGP announcements, can create discrepancies in routing information that are observable from multiple vantage points. The technique of leveraging data from these diverse points can enhance the detection of such forgeries, as inconsistencies in AS path information across different parts of the Internet might indicate a forgery attempt. This criterion supports the inclusion of AS path forgery for its potential to benefit from early detection through a comprehensive global routing table analysis.

AS path forgery does not inherently require collaboration between multiple ASs to be executed. However, the distributed nature of the Internet means that the effects of such forgeries can propagate widely, affecting routing decisions across numerous ASs. While this criterion focuses on collaborative attacks, the widespread impact of AS path forgery suggests that its detection could benefit from group-level analyses, albeit indirectly.

This attack directly involves complex interactions across ASs, as the forged AS path can mislead routers about the best path for traffic, affecting routing decisions globally. Understanding these interactions and the dynamics of AS relationships is crucial for detecting AS path forgery, aligning well with this inclusion criterion. AS path forgery exemplifies the sophisticated manipulation of routing information. Attackers must have a deep understanding of BGP operations and the trust relationships between ASs to craft believable, yet false, AS paths. This level of sophistication in manipulating routing information strongly meets the criteria for inclusion. The nature of AS path forgery allows it to be relatively stealthy, as it can be designed to appear as legitimate routing information. This stealthiness, coupled with the potential for such attacks to evade detection by conventional monitoring systems that may not closely scrutinize AS path attributes, aligns with the criteria for stealthiness and evasive techniques.

### 6.4. AS Path Poisoning

AS path poisoning involves the intentional insertion of AS numbers into the AS path attribute of a BGP announcement to influence route propagation and prevent certain ASs from receiving it. This attack can create discrepancies in routing information observable from multiple vantage points. The technique of leveraging data from diverse points can enhance the detection of such poisoning, as inconsistencies in the AS path information across different parts of the Internet might indicate an attempt at manipulation. This criterion supports the inclusion of AS path poisoning for its potential to benefit from early detection through comprehensive global routing table analyses.

While AS path poisoning itself does not require collaboration between ASs, its impact is distributed across the Internet, affecting routing decisions in multiple ASs. The distributed nature of the impact suggests a benefit from group-level analyses, albeit indirectly, as understanding the propagation of poisoned routes can aid in its detection. AS path poisoning directly involves complex interactions across ASs, as the poisoned AS path can mislead routers about the best path for traffic, affecting routing decisions globally. Detecting this attack requires understanding these interactions and the dynamics of AS relationships, aligning well with this inclusion criterion.

This attack exemplifies the sophisticated manipulation of routing information. Attackers must understand BGP operations and the trust relationships between ASs to craft believable yet strategically poisoned AS paths. This level of sophistication in manipulating routing information strongly meets this criterion for inclusion. AS path poisoning can be relatively stealthy, designed to appear as legitimate routing information while achieving the attacker’s goal of route manipulation. This stealthiness, coupled with the potential for such attacks to evade detection by conventional monitoring systems that may not scrutinize AS path attributes closely, aligns with the criteria for stealthiness and evasive techniques.

### 6.5. Interception Attacks

The strategic impact of interception attacks on routing decisions and data flows is significant, involving the manipulation of BGP attributes and paths to intercept traffic. This manipulation is strategic and requires a nuanced understanding of the BGP’s decision-making processes for its detection and mitigation.

Interception attacks, particularly those executed as adversary-in-the-middle (AitM) operations within the BGP ecosystem, involve diverting and subsequently forwarding traffic to ensure it reaches its intended destination via the attacker’s network. This dual action allows attackers to remain undetected while monitoring or manipulating data. The use of multiple vantage points can enhance the early detection of such attacks by identifying unusual routing patterns or discrepancies in BGP announcements that single-point observations might miss. This criterion supports the inclusion of interception attacks due to the potential benefits of early detection through comprehensive analyses. While interception attacks do not inherently involve collaboration between ASs, their impact and execution can be distributed across the Internet, affecting multiple routing paths and ASs. Our understanding of the distributed nature of these attacks and their propagation could benefit from a group-level analysis, making this criterion relevant for inclusion.

Interception attacks involve complex interactions across ASs, as attackers manipulate BGP routes to insert themselves into the communication path between a source and its intended destination. Detecting these attacks requires an understanding of the dynamics of AS relationships and the ability to analyze routing behavior across multiple points, aligning well with this inclusion criterion.

These attacks exemplify the sophisticated manipulation of routing information, requiring an in-depth knowledge of BGP operations and network topology to execute successfully. The strategic use of BGP announcements to intercept traffic without detection highlights the complexity and sophistication involved, meeting this criterion for inclusion. Interception attacks are characterized by their stealthiness, as they aim to preserve the connectivity and functionality of the network while clandestinely monitoring or manipulating data. This stealthiness, coupled with the ability to evade detection by conventional monitoring systems, aligns with the criteria for stealthiness and evasive techniques.

### 6.6. Replay and Suppression Attacks

These attacks involve the strategic manipulation of the BGP’s operational mechanisms, such as the handling of withdrawal messages and the mitigation of route flapping, rather than the direct falsification of routing information. Understanding these attacks’ effects on the global routing table requires insight into the complex interactions between ASs, and especially how routes are propagated and withdrawn across the network. This supports their inclusion in the analysis. These attacks do not inherently involve collaboration between ASs but can have a distributed impact on the Internet’s routing infrastructure.

While these attacks might not initially seem to benefit from early detection from multiple vantage points, the temporal aspect of replay attacks (retransmitting previously announced routes) and suppression attacks (intentionally delaying or not propagating BGP withdrawal messages) can indeed be better understood and detected with a comprehensive view of routing behavior over time. Grouped AS anomaly detection from multiple vantage points might help identify inconsistencies in route announcements and withdrawals.

### 6.7. Collusion Attacks

Collusion attacks can involve coordinated actions by multiple ASs and the sophisticated manipulation of inter-domain routing principles, such as ASs working together to inject or propagate malicious routing information. The distributed nature of these attacks across different geographic and administrative domains means that leveraging data from multiple vantage points can significantly enhance our detection capabilities. Observing the propagation of malicious announcements from various locations helps in identifying the collaborative pattern of these attacks, making their early detection more feasible.

This criterion directly applies to collusion attacks, as they inherently involve collaboration between two or more ASs. A group-level analysis is crucial for uncovering the coordinated nature of these attacks, making them a prime candidate for detection techniques that analyze interactions and dynamics across multiple ASs. Collusion attacks exploit the trust relationships between ASs to propagate forged or malicious routing information. Understanding these attacks requires a deep analysis of the complex interactions and trust dynamics within the BGP ecosystem. Techniques that can analyze and understand these relationships are well suited to detecting such sophisticated attacks.

The attackers in a collusion scenario use their advanced knowledge of BGP operations and existing trust relationships to manipulate routing information effectively. This manipulation is strategic and requires an in-depth understanding of the BGP’s decision-making processes.

Collusion attacks are designed to be stealthy, evading detection by conventional monitoring systems by appearing to be legitimate BGP announcements from trusted ASs. Our ability to detect these attacks benefits significantly from techniques that can analyze routing data from multiple perspectives to identify anomalies that single-point observations might miss.

### 6.8. MED Modifications and RFD/MRAI Timer Exploitation

MED modification and RFD/MRAI Timer attacks manipulate specific BGP attributes or timers to influence route selection subtly. Both attacks exploit the complex decision-making process of the BGP. Understanding the subtle manipulations of MED or the exploitation of RFD/MRAI timers requires a nuanced understanding of how ASs interact and make routing decisions based on these attributes. These attacks are designed to be stealthy, altering route selection and stability without overt disruptions. Detecting such subtle manipulations benefits from techniques that can aggregate and analyze data from multiple sources, identifying inconsistencies or anomalies in routing behavior that might indicate an attack.

### 6.9. Community Manipulation

Community manipulation, by altering BGP community attributes to influence routing decisions, might benefit from early detection through data from multiple vantage points and group-level AS detection. This manipulation is often subtle and can have widespread effects on routing, making it difficult to detect without comprehensive visibility across different parts of the Internet. Early detection through diverse observations can identify unusual patterns of community attribute usage that deviate from normal behavior.

Community manipulation can involve complex interactions across ASs, as community attributes are used to control routing policies between ASs. Detecting this type of manipulation requires an understanding of how different ASs interpret and act on community values, which can vary widely. Techniques that can analyze these complex interactions are crucial for identifying and mitigating the effects of community manipulation.

This type of attack involves a sophisticated understanding of how BGP community attributes are used within the global routing system to influence routing decisions. Manipulating these attributes to achieve a malicious outcome requires an in-depth knowledge of BGP operations and the policies of various ASs, fitting the criterion for sophisticated manipulation.

Community manipulation is inherently stealthy, as it involves tweaking specific attributes that influence routing decisions without directly altering route paths or AS paths. This subtlety makes it a prime candidate for detection techniques that can aggregate and analyze data from multiple sources, looking for inconsistencies or anomalies that could indicate manipulation.

### 6.10. DoS

In the context of the BGP, a DoS (or DDoS) attack might not directly involve flooding a target with traffic. Instead, it could involve manipulating routing tables to make a network unreachable (blackholing) or redirecting traffic in a way that degrades performance or availability. These actions can be considered as leveraging the BGP to achieve DoS outcomes.

BGP-based DoS attacks, such as those leading to blackholing or unintended traffic redirection, can be sophisticated and involve the strategic manipulation of routing information. However, many DoS examples are also conducted through means beyond the BGP protocol (e.g., the direct flooding of a target’s bandwidth).

### 6.11. Monitor-Aware/Evasive Attack

Monitor-Evasive Advanced Attacks, by design, aim to evade detection by conventional monitoring systems, including public route collectors. These attacks can significantly benefit from early detection through the use of data from multiple vantage points. The ability to leverage diverse observations across the Internet is crucial for identifying these attacks early, before they achieve their malicious objectives. The evasion tactics used in these attacks make them particularly amenable to detection methods that aggregate and analyze data from a wide array of sources to uncover subtle anomalies.

These attacks may involve sophisticated coordination across multiple ASs to ensure their evasion tactics are successful. The distributed nature of these attacks, which aim to remain undetected by selectively targeting or avoiding certain monitors, underscores the need for a group-level analysis that can identify coordinated malicious activities across the network.

Monitor-Evasive Advanced Attacks inherently involve complex interactions across ASs, as attackers must understand the global BGP ecosystem, including the placement and capabilities of monitors, to effectively evade detection. Techniques that can analyze these complex interactions are essential for detecting such advanced evasion tactics. These attacks require an advanced understanding of BGP routing and the operational practices of monitoring systems. Attackers leverage this knowledge to craft attacks that are difficult to detect without analyzing group-level interactions and dynamics, fitting the criterion for sophisticated manipulation.

The hallmark of Monitor-Evasive Advanced Attacks is their use of stealthy maneuvers designed to evade detection by conventional monitoring systems. This criterion is directly applicable to our study, as these attacks manipulate AS path attributes or selectively announce paths to bypass detection, necessitating advanced detection techniques that can identify such evasive maneuvers.

## 7. Survey of Anomaly Detection Techniques

This section provides an in-depth survey of 178 anomaly detection techniques, focusing on their capacity to detect advanced BGP attacks through MVP and parameter scope analyses. These techniques are evaluated based on their computational efficiency, parameter scope, and ability to analyze group-level AS information and dynamics. This review categorizes these approaches into seven distinct groups, including machine learning, deep learning, and signal analysis, identifying potential candidates for next-generation BGP anomaly detection that meet the MVP criteria and are capable of detecting advanced attacks that exploit visibility limitations and complex interactions across multiple ASs.

Prior approaches to determining the characteristics for next-generation BGP anomaly detection (AD) relied upon detecting and identifying the anomaly type and its source [62]. We add MVP as a requirement for next-generation AD and develop a taxonomy for advanced BGP attack detection techniques which is split into seven categories—Classic Machine Learning, deep learning, data mining, outlier detection, signal analysis, statistics, and stochastic learning—in a similar way to how [17] grouped time series anomaly detection methods generally. Previous work has examined whether a technique can handle time series data, uses control plane or data plane data, is univariate or multivariate, can differentiate between types of BGP anomalies, identifies anomaly source networks, and detects attacks in real time [62]. While we do incorporate these criterion into the collection of our sample, we also evaluate these anomaly detection techniques for evidence of the following:

- Their parameter scope;
- Their ability to be deployed using groups of multiple observable ASs;
- Their ability to identify how the peers in a group of ASs are similar or different, how they interact with each other, and extant group-level AS dynamics;
- Their ability to capture and quantify the group interactions, dynamics, and information about collective ASs, with the objective of the group-level high-dimensional MVP anomaly detection of multiple observables (i.e., advanced BGP anomaly detection).

Previous research extensively reviewing hundreds of time series anomaly detection techniques has found that, on average, each algorithm requires the tuning of approximately seven distinct parameters [63,64]. In this context, we define a low parameter scope as ≤2 parameters. Techniques requiring fewer parameters are not only computationally more efficient but also reduce the risk of overfitting, making them more suitable for real-time BGP anomaly detection. Studies on BGP anomaly detection are often dominated by approaches that involve numerous features, parameters, and domain-specific tuning. While these methods may yield high accuracy, they contribute significantly to unacceptable computational costs, which are impractical for deployment in BGP routers. As outlined in Section 4, the impact of additional parameters on the BGP’s routing performance has been well documented. Techniques with a lower parameter scope are necessary to meet the speed and efficiency demands of real-time anomaly detection in BGP networks.

Any approach to BGP anomaly detection possesses strengths and limitations. Detection techniques can be highly accurate, may detect a wide spectrum of anomalies, and may identify the source of an anomaly; yet despite achieving any (or all) of these aspects, they might be limited by speed. For example, some time series approaches using wavelet transforms have shown promise in locating the source of an anomaly; however, they have also proven to be slow [65]. Other studies have sought to identify the source of an anomaly. For example, ref. [66] applied Fast Fourier Transform (FFT) techniques to nine months of BGP data but was unable to identify the source. Two studies using Wavelet techniques (db5 and Haar) successfully identified the source cause [65,67]. Other studies have utilized FFT (among other techniques) for the specific purpose of periodicity identification, with the source cause remaining elusive; the use of an RQA may also be capable of doing so [68]. It is important that any low-parameter MVP technique identified as a candidate for advanced BGP attack detection does not forfeit these previously identified criteria. A highly accurate but computationally expensive technique remains inappropriate for BGP-speaking routers.

We evaluated 41 Classical Machine Learning (ML) approaches to AD in Table 3. Examples of ML techniques are described in [69], citing the previous use of K-means and DBSCAN techniques, whilst the use of a DenStream approach was central to the anomaly detection engine, with later work reporting accuracy metrics of up to 99% [70]. In contrast, previous work using supervised learning (and a Random Forest classifier) reported a 95.71% classification accuracy, although the work had limitations such as an inability to classify forged AS paths if the attacker is a tier-1 or tier-2 AS [10]. Bayesian models have been previously described for the purpose of BGP anomaly classification, such as Naïve Bayes (NB) classifiers [71]. Support Vector Machine (SVM) and Hidden Markov Model (HMM) classifiers have also been used; for example, the use of an SVM for BGP anomaly detection in significant incidents (e.g., Code Red I, Nimda, and Slammer) has been previously described [72]. Interesting work with graph features and a range of algorithms has also been conducted. For example, ref. [73] utilized the PageRank algorithm to develop a novel Ontological Graph Identification (OGI) approach for the detection of hijacks and compromised transit nodes. Ref. [74] was one of the first to both utilize graph features and subsequently explore their use as ML inputs to detect anomalies in the BGP. Of the classic ML approaches evaluated in Table 3, none showed any evidence of being deployed on multiple observables to capture and quantify the group dynamics and information of collective ASs—an essential criterion for group-level high-dimensional MVP anomaly detection to improve advanced BGP attack detection. Some approaches can be used for dimensionality reductions (for example, PCA).

Artificial Neural Network (ANN) models have been used to detect a range of anomalies including Internet blackouts, leaks, and worm attacks [110]. The application of Recurrent Neural Networks (RNNs) for BGP hijack detection has been previously described in the literature [111]. There have been extensive investigations of RNNs and the BGP, such as Long Short-Term Memory (LSTM), Gated Recurrent Unit (GRU), and Broad Learning System (BLS) approaches, which have been previously utilized for their ability to classify time series data [112,113]. As we detail in Section 8, of the 55 evaluations in Table 4, only Federated Learning (FL) has demonstrated some evidence that it could be deployed from multiple observables to capture AS information with the potential for high-dimensional anomaly detection from collective ASs. However, it remains unclear whether FL would capture information on the group dynamics of ASs, identifying how these peers are similar or different, how they interact with each other, and extant group-level AS dynamics.

We evaluated 26 statistical pattern recognition approaches, as shown in Table 5. A number of studies used techniques such as the Exponentially Weighed Moving Average (EWMA), Generalized Likelihood Ratio Test (GLRT), and a Principal Component Analysis (PCA)-powered subspace approach. For example, the use of a PCA-based subspace method with BGP volume extraction was successful in the detection, identification, and differentiation of BGP anomalies, though its router configuration requirements showed that it was prohibitive to real-time detection [161]. PCA has also been combined with EWMA and GLRT with some success [162]. BGP routers have been described as having the characteristics of determinism, periodicity, and recurrence [16,68,163]. Based on these and other similar characteristics, it has been successfully shown that a Recurrence Quantification Analysis (RQA) can detect different types of BGP anomalies in near real time [11,16,68]. We have identified the multidimensional variant of an RQA as a possible candidate for deployment from multiple observables to capture and quantify the group-level interactions, dynamics, and information of collective ASs, with the objective of the group-level high-dimensional MVP detection of advanced BGP attacks. We expand on this assessment in Section 8.

We evaluated 13 papers that used stochastic learning techniques (Table 6). In both the LaserDBN and Multi Hidden Markov Model (MultiHMM) techniques, the anomaly score is formed from a subsequence likelihood index based on probabilistic models. While MultiHMM is also a semi-supervised technique that builds the model from a normal training time series, it was shown to perform well in terms of run-time against unsupervised techniques. The use of HMMs combined with classic ML techniques has been applied to detect BGP anomalies, though they did not appear capable of identifying the location of the source anomaly. Gardiner [185] applied HMMs to BGP anomaly detection, but like all stochastic learning techniques it did not demonstrate any evidence that it would be capable of multiple observable deployments to capture and quantify group-level AS dynamics and information, with the potential for the group-level high-dimensional MVP anomaly detection of advanced monitor-evasive BGP attacks.

There were 10 signal analysis approaches evaluated, five of which were applied to BGP AD, as shown in Table 7. In DWTMLEAD, pre-processing is achieved through a discrete wavelet transform (DWT) and similarly to a MultiHMM approach; the anomaly value is produced from a subsequence log-likelihood [197]. Spectral Residual (SR) and FFT are two unsupervised signal analysis methods that produce an anomaly measure from the discrepancy between reconstructed subsequences and originals. Neither of these have been applied to BGP detection nor do they show any evidence of an advanced MVP BGP attack detection capability. Among the early BGP detection works, ref. [66] is an example of the application of the FFT to BGP detection where neither the anomaly source nor cause could be identified. A DWT with Harr wavelets was used for BGP anomaly detection work in [67] on the research network Abilene, though there were detection delays left unaddressed. A wavelet Daubechies 5 (db5) wavelet transform was used in [65] to identify anomaly origins, though was shown to not be a real-time detection candidate. Wavelet transform techniques were used for BGP detection in [198]. In [199], a Singular Spectrum Analysis (SSA) and the Hilbert Huang Transformation (HHT) were applied to BGP updates, specifically to investigate the Slammer worm incident. From the studies evaluated in Table 7, no possible candidates for deployment from multiple observables to capture and quantify the group-level dynamics and information of collective ASs, with the objective of group-level high-dimensional MVP anomaly detection, were identified.

There were 13 outlier detection techniques evaluated. Several distance-based and nearest neighbour-based methods were evaluated in the outlier detection category, such as local outlier factor (LOF)-based techniques (Table 8). Anomalous activity is identified as irregular subsequences that have large distance metrics from their neighbor. Sub-LOF has demonstrated precision and robustness in the literature, though it has never been applied to BGP anomaly detection [17,204]. Histogram-based Outlier Detection (HBOS) has been used for BGP anomaly detection, in addition to Isolation Forest and CBLOF, and all were similar in their anomaly detection ability [92]. We could not identify any evidence in the outlier detection schemes we evaluated of their capacity to be deployed from multiple observables to capture and quantify the group-level dynamics and information of groups of ASs. Therefore, no technique was evaluated as a candidate for the MVP detection of advanced BGP attacks.

There were 28 data mining anomaly detection techniques evaluated, as seen in Table 9. As with outlier detection, both distance-based and nearest neighbor-based methods feature in data mining (e.g., the Matrix Profile family of algorithms such as HOT SAX, MPA, STAMP, and STOMP). Most of the distance-based methods evaluated were unsupervised. The Matrix Profile (MP) approach has been evaluated on hundreds of time series datasets [63,217] and has been shown to successfully detect anomalies in data with periodic characteristics, with minimal parameterization [218]. A standard MP has been shown to be successful in the detection of BGP anomalies in several incidents [57]. There is no evidence that standard MP algorithms would have the ability to capture and quantify the group-level AS interactions, dynamics, and information with the objective of group-level high-dimensional MVP anomaly detection; however, a multidimensional variant of the MP known as the Discord-Aware Matrix Profile (DAMP) [219] appears to suggest that they can be modified to work with high-dimensional data. However, it remains unclear whether DAMP would capture information on the group dynamics of ASs, such as identifying how their peers are similar or different, how they interact with each other, and the extant group-level AS dynamics. Interesting MP advancements in leader–follower dynamics for the purposes of understanding collective behaviors also represents an avenue for MP to work as a possible MVP BGP anomaly detection technique [220]. We found no evidence that any of the remaining data mining techniques could capture and quantify the group dynamics or information of collective ASs, with the objective of the group-level high-dimensional MVP anomaly detection of multiple observables (i.e., an advanced BGP anomaly detector). The next section assesses some potential candidates for advanced MVP BGP anomaly detection.

## 8. Advanced BGP attack Detection Candidates

This section presents an evaluation of candidate approaches for advanced BGP attack detection, focusing on techniques that can capture group-level AS dynamics and information for MVP anomaly detection. While many anomaly detection techniques are capable of analyzing multivariate data, the requirements for a detection scheme that can detect advanced BGP attacks include an ability to capture group-level AS dynamics and information for purposes of rapid MVP anomaly detection.

Smart BGP attacks are designed to avoid observation by the manipulation of their propagation [15,46]. Research has shown the existing public route collection and monitoring infrastructure is insufficient for advanced BGP attack detection, though conclusions were presented that suggest that more monitors reporting more paths to the route collection infrastructure may improve this. The same research also suggests that future work should investigate the strategic placement of monitors [15,46]. While a monitor-evasive attack is designed to exploit blind spots in public routing infrastructure, the impact of large numbers of collectors on private routing collection infrastructure is untested. For example, Catchpoint has 500 private route collectors for topological-based BGP monitoring purposes. Regardless, an MVP detection scheme would only make this more powerful in the detection of advanced BGP attacks [15,46]. In this section, we summarize the candidate approaches for advanced BGP attack detection and provide an analysis of one of them (multidimensional RQA).

### 8.1. Federated Learning

There are ongoing research efforts to enhance the performance of Federated Learning (FL) models and mitigate communication costs in distributed network environments [244,245]. FL has been used for privacy-preserving route leak detection research, and a technique known as Federated Learning Route Leak Detection (FL-RLD) has been described in the literature [129]. The research showed that ASs with more peers were valuable for route leak detection and that a method using multiple ASs produced a better performance than those using only a single AS observable. For example, the FL-RLD method had better accuracy, precision, recall, and F-score metrics. Whilst [129] studied direct route leak events, it did not consider other BGP incident categories, such as indirect cyber events or outages. This research suggests the importance and value of more peers for improved anomaly detection and may have the potential for higher-dimensional capabilities. It remains unclear whether the model amalgamation processes underlying FL achieve the criteria specified in Section 7; there was no evidence identified in the surveyed literature that FL can capture and quantify group-level interactions and dynamics information for the anomaly detection of collective ASs. There is also complexity associated with tuning FL’s multiple parameters that might hinder its adaptability and swift deployment in dynamic network environments, though this requires further research.

### 8.2. Multidimensional and Leader–Follower MP

Multidimensional variants of the MP technique represent areas for future research on MVP BGP anomaly detection. Unlike a majority of time series detection algorithms, MP is unfazed by large, sparse datasets. It allows for anytime computation whilst being extremely scalable and storage-efficient; massive datasets can be processed in its main memory, for example, and MP is extremely parallelizable. Due to an exceptionally low parameter scope, MP discords minimize overfitting and are also free of data assumptions. MP has also shown that it can discover anomalies in datasets with missing data, with no FNs [57,246,247].

A variant of MP for motif discovery (repeated patterns) in multidimensional data was described in [248], though further research would be required to ascertain whether there is any utility to its multidimensional discord discovery. Additionally, DAMP provides some evidence that it can work with high-dimensional data, such as that from group-level AS information [219]. For example, it appears that this modified form could accommodate multidimensional data from multiple sources. Although there was no evidence in the literature that this technique can capture information on the group-level interactions and dynamics of multidimensional systems, this is an avenue for further work to explore.

Interesting MP advancements in leader–follower dynamics for the purposes of understanding collective behaviors also represent an avenue for MP as a possible MVP BGP anomaly detection technique [220]. This approach leverages MP to identify leader and follower patterns within time series data, offering a potential candidate for capturing the dynamics of collective behaviors across ASs at the group level.

**Figure 10:**
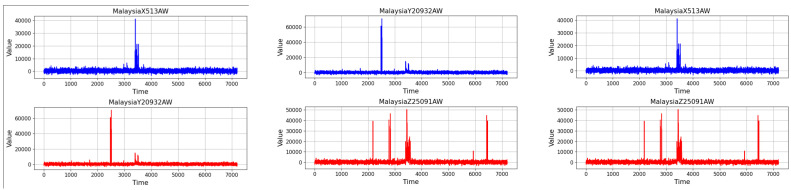
MP leader–follower dynamics for BGP detection.

Preliminary results from applying this technique to the Telekom Malaysia incident suggest its effectiveness in capturing leader–follower motif patterns among ASs (Figure 10 illustrates the application of MP leader–follower dynamics in the Telekom Malaysia incident). By identifying motifs and discords within AS interactions, this method holds promise for unveiling the intricate dynamics that underpin collective anomalies in BGP data. However, these initial findings necessitate further validation through comprehensive analyses to determine if leader–follower discords are possible and to validate MP’s utility as a collective AS BGP detection technique.

### 8.3. Multidimensional RQA

As noted in previous sections, BGP routers have been successfully modeled as nonlinear dynamical systems and have the characteristics of determinism, periodicity, and recurrence. Based on these and other similar characteristics, it has been shown that an RQA can detect different types of BGP anomalies [11,16,68]. Standard RQA has been established as an effective near-real-time anomaly detection metric, but reported similar limitations as all methods deployed from a single observable. However, multidimensional RQA (MdRQA) is a candidate that can potentially be deployed to capture collective AS interactions and dynamics information for the purpose of MVP anomaly detection, and one that provides a quantitative multidimensional technique for the dynamical system that is BGP and the complex environment that is the Internet. MdRQA is assumption-free and has been shown to be robust and non-stationary in outlier challenges. While standard RQA measurements have shown indications of a detection capability, they have limitations [68].

MdRQA is an established technique that allows researchers to investigate how groups differ from one another in terms of their dynamics [166]. As the multidimensional variant of RQA, the use of MdRQA for groups involves embedding multiple time series into a phase space. Whilst other correlation variants exist, it has been shown that such techniques are not capable of capturing important information and dynamics at the system level [166,249]. This simple correlation of dyadic relationships does not capture the truth of the dynamics of the group. MdRQA can capture and quantify higher-dimensional dynamics, which drives our hypothesis about BGP anomaly detection.

We posit that the use of MdRQA may improve standard RQA measurements for the purposes of capturing group dynamics and information, as shown in previous work with groups of people [166]. The Recurrence Rate (RR) is the probability that the system recurs (e.g., the density of recurrence) (Equation (1)). The determinism measurement (DET) is a predictability measure based on the diagonal lines of recurrence points and the percentage of recurrence points that form those structures (Equation (2)). Lines will differ depending on the system. For example, chaotic systems will produce shorter lines and periodic systems longer lines. The maximum length (MaxL) is that of the diagonal structure formed by adjacent recurrent points (Equation (3)). The average length of these diagonal structures (MeanL) is formed by their recurrent points (i.e., the mean time trajectory segments are close to each other) (Equation (4)). We leave a rigorous examination of all other RQA measurements to future work and summarize the metrics used in this exploratory work as follows:

- The Recurrence Rate (RR) is the probability that the system recurs.
  (1) $$RR=\frac{1}{N^{2}}\sum_{i,j=1}^{N}R_{ij},$$
- The determinism measurement (DET) is a predictability measure based on the diagonal lines of recurrence points and the percentage of recurrence points that form those structures.
  (2) $$DET=\frac{\sum_{l=l_{\min}}^{N}l\;P\left(l\right)}{\sum_{i,j=1}^{N}R_{ij}},$$
- The maximum length (MaxL) of the diagonal structure formed by adjacent recurrent points.
  (3) $$MaxL=\max(l_{i};i=1,\cdots ,N_{l}),$$
- The average length of the diagonal structures (MeanL) formed by recurrent points or the mean time trajectory segments that are close to each other.
  (4) $$MeanL=\frac{\sum_{l=l_{\min}}^{N}l\;P\left(l\right)}{\sum_{l=l_{\min}}^{N}P\left(l\right)},$$

It is hypothesized that anomaly detection metrics are improved by analyzing ASs as groups in higher dimensions, in contrast to inferring multidimensional dynamics from single observables. As MdRQA captures the multidimensional dynamics of a group of ASs, this may allow for high-dimensional anomaly detection with higher fidelity. Previous work has shown that MdRQA captures the higher fidelity dynamics of a system (e.g., time series of *X*, *Y*, and *Z* concurrently) as opposed to s standard (unidimensional) RQA, which is based on an approximation of a single dimension (e.g., *X*, or *Y*, or *Z*) [166]. In contrast to a standard RQA, its multidimensional variant can incorporate multiple observables to be used as dimensions in the phase space, obtained from the group (or group of systems) being analyzed. While the technique has been successful in quantifying the group dynamics of people, it has never been applied to groups of computer-controlled devices before now.

Imagining the same busy city mall, where people represent ASs and their interactions symbolize BGP messages, we can further explore the dynamics using the scenario of Mallory, a malicious character planning to disrupt the flow of people within the mall. Mallory, representing a malicious AS (ASM), spreads false information about a celebrity giveaway, analogous to a BGP hijack where a false IP prefix is announced.

Alice (AS1) immediately travels towards the supposed event, representing an increase in BGP announcements. Alice’s heart rate increases while texting friends about the news, similar to an increase in BGP volume. Bob (AS2), skeptical, continues on his usual path, representing stability in the AS path length. Carol (AS3) verifies the information, experiences physiological changes, and checks with others before deciding to change her route, analogous to BGP withdrawals and re-announcements.

**Figure 11:**
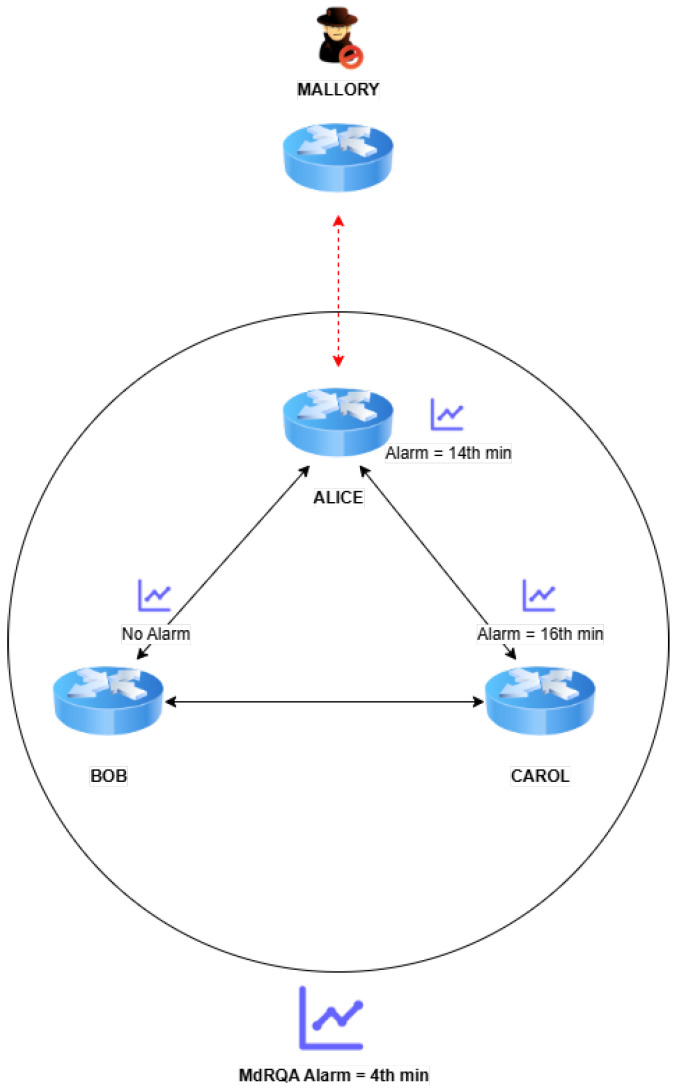
MdRQA group anomaly detection.

Using a standard RQA, individual reactions provide us with specific insights (Figure 11); Alice’s actions signal an anomaly through increased announcements and volume, Carol’s cautious behavior shows withdrawals and re-announcements, and Bob’s stable path shows no noticeable anomalies. However, the standard RQA misses subtle changes and the broader pattern of group interactions.

Using MdRQA, the focus shifts to collective dynamics. Alice’s reaction influences Bob and Carol, causing a commotion that catches Bob’s attention. MdRQA captures these subtleties, detecting anomalies much earlier by analyzing collective behaviors. The method reveals hidden patterns and connections, enabling the more effective detection of advanced BGP attacks.

MdRQA provides a comprehensive understanding of how misinformation (a BGP hijack) propagates through the network (mall). Anomalies are detected by analyzing group dynamics, capturing their collective impact rather than isolated incidents. This approach quantifies group information and interactions, leading to earlier anomaly detection.

In the context of the BGP, MdRQA allows for the simultaneous observation and analysis of multiple ASs, capturing the complex interplay between them. This multi-observational capacity is crucial, reflecting the reality of BGP operations in a complex, distributed system. Addressing visibility limitations is increasingly important, given recent descriptions of advanced BGP attacks that exploit these vulnerabilities [15].

**Figure 12:**
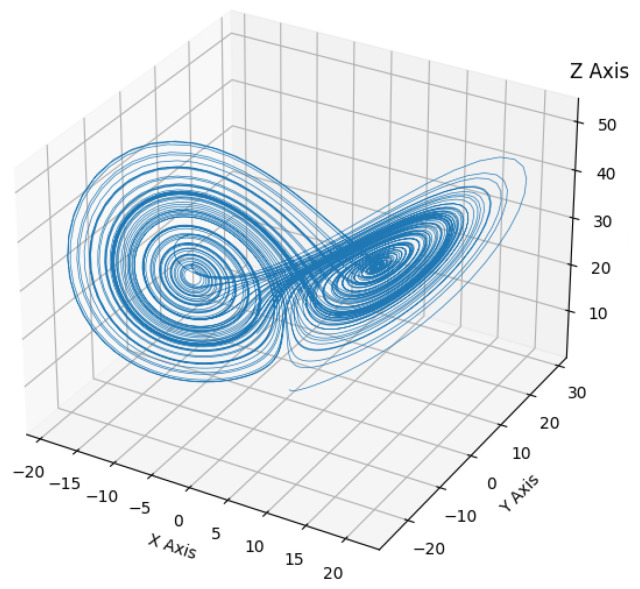
Trajectory in phase space of Lorenz Attractor.

MdRQA is capable of capturing the dynamics of a single multidimensional system and capturing information on the group dynamics between different systems [166,249]. Consider the Lorenz Attractor (Figure 12) as an example where the multidimensional dynamics of a single chaotic multidimensional system can be inferred from a single observable [166]. For example, the diagonal line structures and metrics of MdRQA, such as the average diagonal line length (MeanL) and longest diagonal line length (MaxL), are shown to be longer for the MdRQA in contrast to RQA metrics [166]. RQA metrics can be consistently improved when a system is quantified in contrast to a single observable (Table 10). This is an example of MdRQA’s capacity to incorporate multiple observables as opposed to inferring the dynamics from a single observable. Table 10 shows that MdRQA quantifies the system’s dynamics and outperforms a standard RQA in the majority of the RR, DET, MeanL, and MaxL metrics.

## 9. Discussion and Future Work

Advanced BGP attacks such as monitor-evasive attacks [15] exploit the vulnerabilities inherent within public Internet collector infrastructure, which are exacerbated by the limited visibility of vantage points. BGP anomaly detection research is almost exclusively dominated by techniques that collect public route collector data from a single observable or monitoring point. Current solutions are reliant on distributed route collectors, such as those facilitated by public route collectors (i.e., RIPE and Routeviews). However, the extant research on this form of attack has not considered the private collector infrastructure and this is left for future work. Nevertheless, regardless of whether an anomaly detection scheme is deployed from public or private infrastructure, the detection of advanced BGP attacks will require a scheme that can capture the dynamics of groups of ASs for high-dimensional MVP anomaly detection. Capturing and investigating the interactions among groups of ASs, be they from public or private collections and monitoring infrastructure, can result in a powerful anomaly detection technique that can mitigate the visibility limitations exploited by advanced BGP attacks.

While several techniques met one of the MVP criteria outlined in Section 7, only MdRQA has shown evidence that it could identify how the peers in a group of ASs are similar or different and how they interact with each other and capture and quantify group-level AS dynamics and information on collective ASs, with the objective of the high-dimensional MVP anomaly detection of multiple observables (i.e., an advanced BGP anomaly detector). In summary, MdRQA is capable of capturing both the dynamics of a single multidimensional system and capturing information on the group dynamics between different multidimensional systems.

Of the candidates identified for advanced BGP anomaly detection, and to our knowledge, MdRQA has also never been applied to computer-controlled systems before. This warrants further work to investigate whether MdRQA can be developed into an MVP BGP anomaly detection scheme.

## 10. Conclusions

The Internet is a complex environment. Advanced attacks exploit the Internet’s complexity. To date, most approaches to BGP anomaly detection have been almost entirely investigated using public collector infrastructure and from single observables, which can be used to monitor an AS from a single monitoring point.

Investigating how the peers in a group of ASs are similar or different, how groups of ASs interact, and capturing their group dynamics can provide a powerful approach to BGP anomaly detection. This requires a technique that can not only be deployed across multiple viewpoints, capturing information about the interaction of multiple peers in a collector, but also one that can quantify group dynamic information and high-dimensional anomalous activity.

We posited that next-generation BGP detection will require the capacity to capture group-level dynamics, interactions, and information from ASs to quantify and encapsulate multi-viewpoint information. We evaluated 178 anomaly detection techniques and identified potential candidates for advanced BGP attack detection. We conducted an exploratory study of two candidate techniques, Matrix Profile and Multidimensional RQA, with promising results. 

## Figures and Tables

**Table 1:** Inclusion and exclusion criteria for evaluating BGP attacks.

Criteria Type	Description
Inclusion 1	Attacks that could be detected earlier by leveraging data from multiple vantage points across the Internet, providing a more comprehensive view of the global routing table than single-point observations. This includes attacks where discrepancies in BGP announcements across different locations could indicate an anomaly, requiring the correlation of data from multiple sources for early detection (attack surface and temporal elements).
Inclusion 2	Attacks that involve collusion between ASs or are distributed in nature, benefiting from a group-level analysis to uncover coordinated malicious activities (collaborative or distributed nature).
Inclusion 3	Attacks that involve complex interactions across multiple Autonomous Systems, especially those that require an understanding of the dynamics of AS relationships to be detected (complex interactions across ASs).
Inclusion 4	Attacks that involve the advanced manipulation of routing information, where attackers leverage in-depth knowledge of the BGP to craft attacks that are difficult to detect without analyzing group-level interactions and dynamics (sophisticated manipulation of routing information).
Inclusion 5	Attacks that employ stealthy maneuvers or aim to evade detection by conventional public inter-domain routing monitoring and collector infrastructure. This includes attacks that manipulate AS path attributes or selectively announce paths to bypass detection by public route collectors (stealthiness and evasive techniques).
Exclusion 1	Attacks that are direct and lack complexity, such as simple prefix hijacking without any evasion tactics, might not benefit as much from a multi-vantage point approach since they can often be detected by conventional means (direct, simple attacks).
Exclusion 2	Attacks that do not directly involve BGP manipulation and predominantly rely on vulnerabilities outside the BGP protocol itself (non-BGP layered attacks).
Exclusion 3	Attacks whose impact on routing decisions is not strategic or does not involve the manipulation of BGP attributes or paths. This includes attacks that, while they may cause disruption, do not require an understanding of the BGP’s decision-making process or the relationships between ASs to be detected or mitigated.

**Table 2:** Evaluation of BGP attacks in terms of these criteria.

Attack Type/Criterion	I1	I2	I3	I4	I5	E1	E2	E3
Prefix Hijacking	Y	N	N	N	N	Y	N	N
Subprefix	Y	N	Y	Y	Y	Y	N	N
AS Path Forgery	Y	N	Y	Y	Y	N	N	N
AS Path Poisoning	Y	N	Y	Y	Y	N	N	N
Interception Attacks	Y	N	Y	Y	Y	N	N	N
Replay and Suppression	Y	N	Y	N	N	N	N	N
Collusion Attack	Y	Y	Y	Y	Y	N	N	N
MED and RFD/MRAI	Y	N	Y	Y	Y	N	N	N
Community Manipulation	Y	N	Y	Y	Y	N	N	N
Denial-of-Service (DoS)	P	N	P	P	P	P	P	P
Monitor Evasive	Y	Y	Y	Y	Y	N	N	N

Note: Yes = Y, No = N, Partially = P.

**Table 3:** Classic machine learning.

Citation	Technique	AD	MVP	#Params
[74,75,76,77,78,79,80,81,82]	SVM and its variants	Y	N	>2
[83]	NetworkSVM	N	N	>2
[84]	PhaseSpace-SVM	N	N	>2
[85]	Eros-SVMs	N	N	>2
[86]	ELM, KNN, NB	Y	N	>2
[9,10,73,87]	K-means/DBscan and variants	Y	N	>2
[88]	K-Means clustering	Y	N	>2
[89]	K-Means	N	N	>2
[90]	Hybrid K-Means	N	N	>2
[91]	HMM and Tukey’s	Y	N	>2
[92]	HBOS and others	Y	N	≤2
[32,93]	PCA	Y	N	≤2
[94]	RobustPCA	N	N	≤2
[95,96]	Winnowing	Y	N	≤2
[69]	DENSTREAM	Y	N	>2
[97]	c4.5	Y	N	>2
[98]	MS-SVDD	N	N	>2
[99]	sequenceMiner	N	N	>2
[100]	NoveltySVR	N	N	>2
[101]	SLADE-MTS	N	N	>2
[102]	HBOS	N	N	≤2
[103]	PCC	N	N	≤2
[104]	KNN	N	N	≤2
[105]	SLADE-TS	N	N	>2
[106]	XGBoost and variant	N	N	>2
[107]	Adaptive One-Class SVM	N	N	>2
[108]	RUSBoost	N	N	>2
[109]	OC-KFD	N	N	>2

BGP anomaly detection = AD, multi-viewpoint = MVP, Yes = Y, No = N, Possibly = P.

**Table 4:** Deep learning.

Citation	Technique	AD	MVP	#Params
[13,75,78,81,111,112,114,115,116]	LSTM and variants	Y	N	>2
[117,118,119]	Other LSTM variants	N	N	>2
[110]	Deep ANN	Y	N	>2
[120,121]	Deep Embedding Models	Y	N	>2
[122,123,124,125]	RNNs	Y	N	>2
[126]	GAT	Y	N	>2
[127]	GRU	Y	N	>2
[128]	DLAE	Y	N	>2
[129]	**Federated Learning**	**Y**	**P**	**>2**
[130]	Deep Belief Network (DBN)	Y	N	>2
[131]	Normalizing Flow	N	N	>2
[132]	DeepAnT	N	N	>2
[133]	STORN	N	N	>2
[134,135,136,137]	ESNs	N	N	>2
[138]	DeepNAP	N	N	>2
[139]	DANN	N	N	>2
[140]	MTLED	N	N	>2
[141]	Hybrid KNN	N	N	>2
[142]	Hybrid DAE	N	N	>2
[143]	ELM-HTM	N	N	>2
[144]	TCN-AE	N	N	>2
[145]	LTI	N	N	>2
[146,147]	VarAE	N	N	>2
[148]	OMES/MTAD-GAT	N	N	>2
[149]	HTM/RADM	N	N	>2
[150]	MSCRED	N	N	>2
[151]	MEGA	N	N	>2
[152]	Hybrid ARIMA-WNN	N	N	>2
[153]	DL image-based	N	N	>2
[154]	GAN-based	N	N	>2
[155]	Hybrid VAELSTM	N	N	>2
[156]	D^2^S	N	N	>2
[157]	GC-ADS	N	N	>2
[158]	XceptionTimePlus/Telemanom	N	N	>2
[159]	HS-VAE	N	N	>2
[160]	FluxEV	N	N	>2

**Table 5:** Statistical methods.

Citation	Technique	AD	MVP	#Params
[30,40,162]	EWMA and variants	Y	N	≤2
[164,165]	Other EWMA variants	N	N	≤2
[163]	RQA	Y	N	>2
[166]	**MdRQA**	**N**	**Y**	>**2**
[167,168]	Kalman Filter	Y	N	>2
[169]	SARIMA	Y	N	>2
[170]	NIDES/STAT	Y	N	>2
[171]	Heuristic algorithms	Y	N	>2
[172]	Z-score	Y	N	≤2
[173]	MRCD	N	N	>2
[174]	MEDIFF	N	N	>2
[175]	ARFIMA/Holt-Winter	N	N	>2
[176]	MGDD	N	N	>2
[177]	One-sided Median	N	N	≤2
[178]	Seasonal-Hybrid ESD	N	N	>2
[179]	ANODE/R-ANODE	N	N	>2
[180]	RePAD2	N	N	>2
[181]	AMD	N	N	>2
[182]	DCDSPOT	N	N	>2
[183]	SASE/SMSE	N	N	>2
[184]	PCI	N	N	≤2

**Table 6:** Stochastic learning.

Citation	Technique	AD	MVP	#Params
[185]	HMM	Y	N	>2
[186,187,188,189,190,191,192]	HMM variants	N	N	>2
[193,194]	DBNs	N	N	>2
[195]	Interactive OD	N	N	>2
[196]	FABDNBC	N	N	>2

**Table 7:** Signal analysis.

Citation	Technique	AD	MVP	#Params
[66,200]	FFT	Y	N	≤2
[199]	SSA, HHT	Y	N	>2
[201]	DWT	Y	N	>2
[67]	DWT and Haar wavelets	Y	N	>2
[65]	db5 transform	Y	N	>2
[198]	Wavelet transform	Y	N	>2
[202]	SR	N	N	>2
[203]	DWT-MLEAD	N	N	>2
[197]	Online DWTMLEAD	N	N	>2

**Table 8:** Outlier detection.

Citation	Technique	AD	MVP	#Params
[92]	HBOS, Isolation Forest	Y	N	>2
[205]	LOCI, LOF	Y	N	>2
[206,207,208]	LOFs and RFCOF	N	N	>2
[209]	Semi-supervised HIF	N	N	>2
[210,211,212]	IFs	N	N	>2
[213]	COPOD-IKDM	N	N	>2
[214]	Distance-based OD	N	N	>2
[215]	ADSTREAM	N	N	>2
[216]	ATAD	N	N	>2

**Table 9:** Data mining.

Citation	Technique	AD	MVP	#Params
[170]	NIDES/STAT	Y	N	>2
[221]	HOPA	Y	N	>2
[222]	Random Walk	Y	N	>2
[57,63,217,219,223,224,225,226,227,228]	**MP and variants**	**Y**	**P**	≤**2**
[229]	SequenceGram	N	N	>2
[230]	THLS G-GECM	N	N	>2
[231]	OMABD	N	N	>2
[232]	GraphAn	N	N	>2
[233]	ParalellDadd	N	N	>2
[234]	AR Mining	N	N	>2
[235]	GrammarViz3.0	N	N	>2
[236]	NormA	N	N	>2
[237]	OBN-based	N	N	>2
[238]	NP-AP	N	N	>2
[239]	STARE	N	N	>2
[240]	InfoMiner	N	N	>2
[241]	EnsembleGI	N	N	>2
[242]	DADS	N	N	>2
[243]	Weighted-PST	N	N	>2

**Table 10:** RQA and MdRQA measurements of Lorenz Attractor.

	RQA(x)	RQA(y)	RQA(z)	MdRQA
RR	0.69	0.84	0.68	0.69
DET	99.4	97.4	99.5	99.9
MeanL	9.12	7.84	10.3	16.4
MaxL	131	118	82	167
